# EMX2-GPR156-Gαi reverses hair cell orientation in mechanosensory epithelia

**DOI:** 10.1038/s41467-021-22997-1

**Published:** 2021-05-17

**Authors:** Katie S. Kindt, Anil Akturk, Amandine Jarysta, Matthew Day, Alisha Beirl, Michaela Flonard, Basile Tarchini

**Affiliations:** 1 Section on Sensory Cell Development and Function, National Institute on Deafness and Other Communication Disorders, National Institutes of Health, Bethesda, MD USA; 2 The Jackson Laboratory, Bar Harbor, ME USA; 3 Department of Medicine, Tufts University, Boston, MA USA; 4 Graduate School of Biomedical Science and Engineering (GSBSE), University of Maine, Orono, ME USA

**Keywords:** Cell polarity, Morphogenesis, Development, Cochlea, Sensory processing

## Abstract

Hair cells detect sound, head position or water movements when their mechanosensory hair bundle is deflected. Each hair bundle has an asymmetric architecture that restricts stimulus detection to a single axis. Coordinated hair cell orientations within sensory epithelia further tune stimulus detection at the organ level. Here, we identify GPR156, an orphan GPCR of unknown function, as a critical regulator of hair cell orientation. We demonstrate that the transcription factor EMX2 polarizes GPR156 distribution, enabling it to signal through Gαi and trigger a 180° reversal in hair cell orientation. GPR156-Gαi mediated reversal is essential to establish hair cells with mirror-image orientations in mouse otolith organs in the vestibular system and in zebrafish lateral line. Remarkably, GPR156-Gαi also instructs hair cell reversal in the auditory epithelium, despite a lack of mirror-image organization. Overall, our work demonstrates that conserved GPR156-Gαi signaling is integral to the framework that builds directional responses into mechanosensory epithelia.

## Abstract (web summary)

Sensory hair cells develop an asymmetric architecture to restrict stimulus detection to a single axis. Here the authors identify GPR156 as directing a 180-degree reversal in hair cell orientation through Gαi, downstream of EMX2 in the mouse inner ear and zebrafish lateral line.

## Introduction

Hair cells (HCs) are mechanoreceptors that capture sound in the cochlea, head movements in vestibular organs and water movements in the fish lateral line. HCs detect these stimuli through their apical hair bundle. Both proper morphology and orientation of hair bundles within sensory organs are critical for organ function. Previous work showed that inactivating guanine nucleotide-binding proteins of the inhibitory alpha class (Gαi1, Gαi2, Gαi3; collectively Gαi) can impact both hair bundle morphology and HC orientation. Although Gαi regulators required for hair bundle morphology have been identified, how Gαi signals to control HC orientation is not known.

**Fig. 1:**
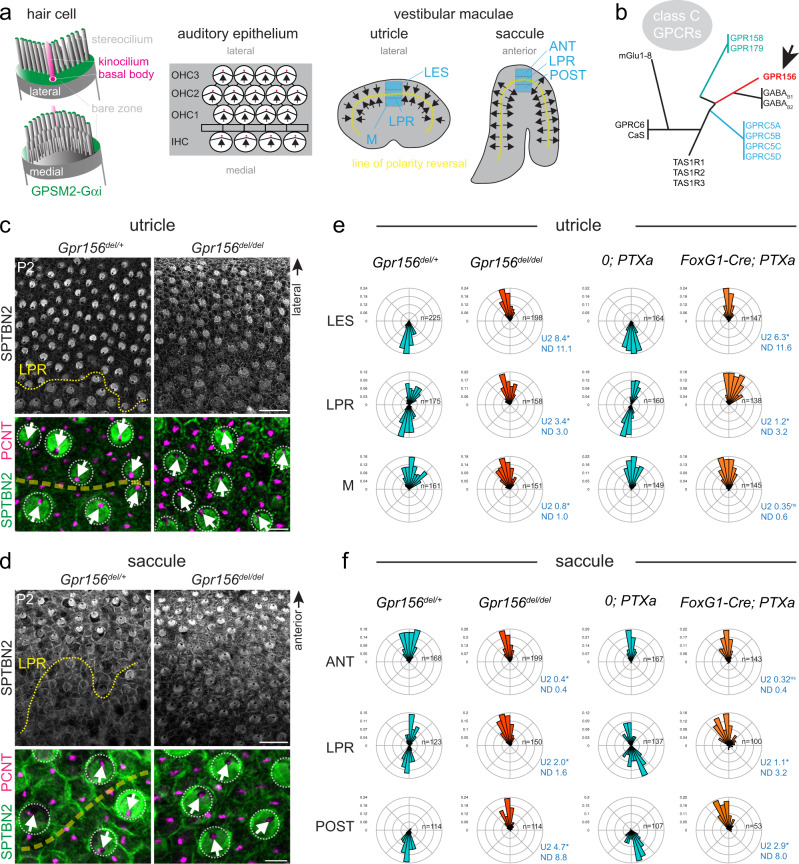
*Gpr156* or Gαi inactivation abrogates hair cell reversal in the mouse maculae. **a** Schemes representing a single auditory HC from the lateral/abneural (top) or medial/neural (bottom) side, and HC orientation (arrows) in the auditory epithelium and the utricular and saccular maculae. In the maculae, two HC populations of opposing orientations are separated by a virtual line of polarity reversal (LPR, yellow dashed line). Domains where HC orientation was quantified in **e**, **f** are indicated in blue (utricle: lateral extrastriolar (LES), LPR and medial (M) domains; saccule: anterior (ANT), LPR and posterior (POST) domains). **b** Phylogenetic tree of class C GPCRs adapted from^62^. **c**, **d** LPR region in P2 utricle (**c**) and saccule (**d**). Top panels show a low magnification view with SPTBN2 (βII-spectrin) labeling revealing HC orientation by the position of the off-center fonticulus devoid of signal. Bottom panels show a distinct region at higher magnification where PCNT (Pericentrin) labels the basal body below the fonticulus. The LPR can be traced in controls but not in mutants, where all HCs generally point laterally in the utricle and anteriorly in the saccule. **e**, **f** Circular histograms of HC orientation by region in the utricle (**e**) and saccule (**f**). Histograms show frequency distribution at P0-P2 (10° bins in a referential where 90° (top) is lateral in the utricle and anterior in the saccule; n indicates HC number in *N* = 4 animals; Watson U2 test of homogeneity; normalized difference (ND) value indicates how many standard deviations separate the circular means of each distribution). *PTXa* indicates the Cre-inducible *R26-LSL-PTXa* allele. Littermate controls for *FoxG1-Cre*; *PTXa* are Cre-negative *PTXa* animals. Arrows indicate HC orientation. Scale bars are 20 µm (**c**, **d** top), 5 µm (**c**, **d** bottom).

At the single HC level, the hair bundle is a brush of actin-based protrusions, or stereocilia, aligned in rows of graded heights (Fig. 1a). This asymmetric morphology enables mature HCs to detect mechanical stimuli in a directional manner—planar deflections towards the tallest row tension extracellular links between rows, opening mechanosensory channels^1^. The asymmetric morphology of the hair bundle originates from the early polarization of the apical HC cytoskeleton along the epithelial plane^2^. When HCs are still covered with microvilli, the primary cilium (kinocilium) and its nucleating basal body shift off-center^3,4^. Gαi and one binding partner, the scaffolding protein GPSM2/LGN, then generate a new region of apical membrane devoid of microvilli on the basal body side, the ‘bare zone’ (Fig. 1a)^5–7^. GPSM2-Gαi helps define the edge of the forming hair bundle as microvilli near the bare zone thicken and grow into stereocilia. Later, GPSM2-Gαi is selectively trafficked to the tips of stereocilia abutting the bare zone, conferring their row 1 tallest identity^8–11^. In canonical signaling, Gαi is a subunit of the heterotrimeric Gαi(GDP)βγ complex that transiently dissociates into active Gαi(GTP) and Gβγ upon GPCR activation to signal to downstream effectors^12^. In contrast, GPSM2 is known to sequester and enrich Gαi in its GDP state^13,14^. This non-canonical GPSM2-Gαi complex has been best characterized in another cell polarity context, the orientation of the mitotic spindle^15,16^. Thus, non-canonical Gαi activity promotes stereocilia placement and elongation to establish the asymmetric hair bundle morphology required for a directional HC response.

At the organ level, neighboring HCs coordinate the orientation of their asymmetric apical cytoskeleton, including the hair bundle, along the epithelial plane to mount a coherent response to sensory stimuli. This organization relies on core planar cell polarity (PCP) proteins that relay orientation information via intercellular interactions^2,17,18^. PCP proteins are asymmetrically enriched at apical junctions between HCs and adjacent support cells, and ensure for example that the one row of inner HCs (IHCs) and 3 rows of outer HCs (OHCs) adopt a uniform lateral/abneural orientation in the auditory epithelium (Fig. 1a). In contrast, in vestibular otolith organs (the utricle and saccule maculae)^19–21^ and neuromasts in the fish lateral line^22,23^ this uniform HC orientation is broken. These organs have two HC populations with opposing orientations that align along a line of polarity reversal (LPR; Fig. 1a). This mirror-image anatomy allows maculae and neuromasts to detect stimuli in a bidirectional manner^24–26^.

Recent work found that the transcription factor EMX2 breaks the uniform orientation defined by core PCP proteins in mouse maculae and zebrafish neuromasts^27–31^. *Emx2* is regionally expressed in just one HC population (Fig. 1a), and functions to reverse its orientation by 180°. In both systems, loss of EMX2 abrogates the LPR so that all HCs are uniformly oriented. Gαi also participates in HC orientation reversal, as inactivating Gαi with pertussis toxin (PTX) partially prevents EMX2^+^ macular HCs from reversing their orientation^27^. Intriguingly, inactivating Gαi in auditory HCs that all express *Emx2*^27^ not only disrupts stereocilia placement and elongation, but also inverts OHC orientation in a graded manner across rows^5,8^. As OHC orientation defects were not observed in *Gpsm2* mutants^5–7^, Gαi must work with a different regulator to instruct HC orientation reversal.

Here we hypothesized that canonical Gαi signaling downstream of a GPCR instructs HC orientation reversal. We ascribe a function to GPR156/GABABL, an orphan class C GPCR with high homology to the GABA_B_ metabotropic receptors (GABBR1-GABBR2)^32–34^. We find that GPR156 is planar polarized by EMX2 and signals through Gαi to trigger HC orientation reversal. We show that GPR156-Gαi is essential to generate mirror-image HC organization in otolith organs and in neuromasts, where Gpr156 enables detection of bidirectional fluid currents. Finally, we demonstrate that GPR156-Gαi is required for uniform HC orientation in the cochlea and for auditory function. Overall, this work identifies a conserved membrane receptor required to reverse HC orientation, a developmental process that proves equally critical for hearing, balance, and sensing water movements.

## Results

### No hair cell reversal upon GPR156 or Gαi inactivation

**Fig. 2:**
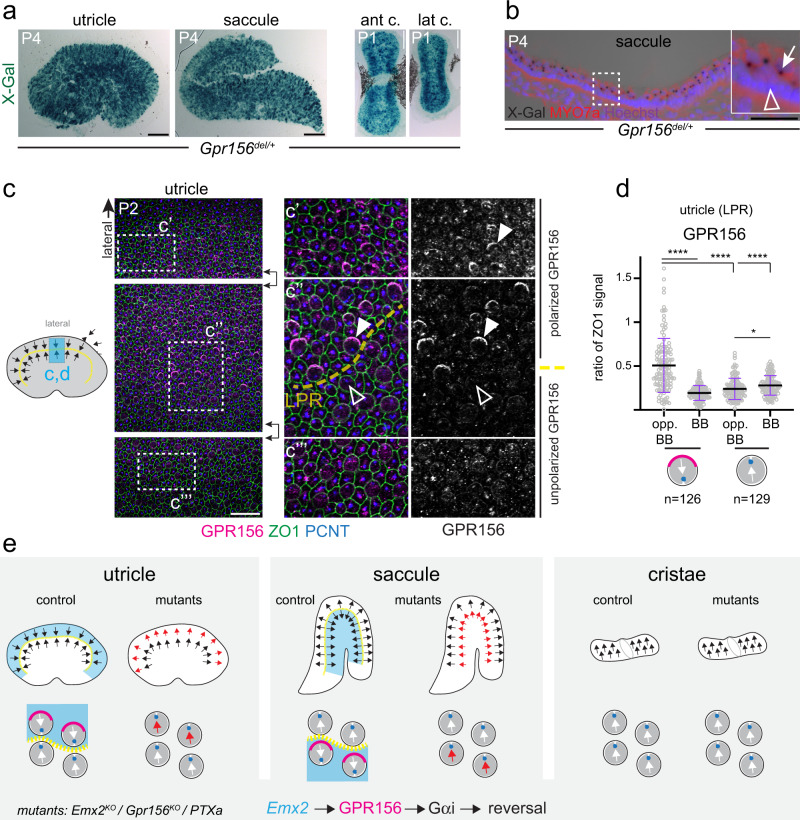
*Gpr156* expression and GPR156 protein localization in mouse vestibular organs. **a** *LacZ* reporter is expressed throughout the sensory region in *Gpr156*^*del/+*^ vestibular organs (ant/lat c., anterior/lateral crista). **b** *LacZ* expression is limited to MYO7A^+^ HCs in a saccule cross-section. X-gal signal is trapped in HC vesicles (arrow in magnified inset) but support cells (arrowhead) are negative. **c**, **d** P2 wild-type utricle where basal body labeling (PCNT) indicates HC orientation. GPR156 polarization (solid arrowheads) is limited to lateral HCs oriented medially. HCs across the LPR oriented laterally do not show polarized GPR156 (hollow arrowheads). Boxed regions in continuous fields in the left panels are magnified in the central and right panels (saccule: see Supplementary Fig. 2c). **d** GPR156 enrichment in the utricle LPR domain at the HC junction opposite (opp. BB) or near (BB) the basal body. HCs oriented medially (left) are analyzed separately from HC oriented laterally (right). GPR156 is expressed as ratio of ZO1 signal (mean ± SD; *n*, HC numbers in 3 animals; Kruskal-Wallis test with Dunn’s multiple comparisons, ****p < 0.0001; *p = 0.0332). **e** Summary of HC orientation (arrows), GPR156 protein distribution (magenta) and previously reported *Emx2* expression (blue) by vestibular organ in normal and mutant conditions. The scheme in **c** indicates the position of the domain analyzed in **c** and **d** (blue). Scale bars are 100 µm (**a**), 50 µm (**b**), 20 µm (**c**).

HC orientation reversal is a hallmark feature of otolith organs in the vestibular system. In the mouse utricular and saccular macula, *Emx2*^+^ HCs reverse their orientation to create a virtual line of polarity reversal (LPR) that bisects the organ (Fig. 1a)^27^. We used βII-spectrin (SPTBN2) or pericentrin (PCNT) labeling to reveal the position of the off-center basal body to determine vestibular HC orientation. We found that globally inactivating Gαi function through expression of Pertussis toxin catalytic subunit (PTXa) led to a complete loss of HC reversal in the maculae, effectively abrogating the LPR (*FoxG1-Cre;* PTXa^8^; see Figs. 1e, f and 2e). This result confirms that Gαi activity is required for HC reversal, as previously suggested^27^ based on incomplete loss of HC reversal in a less potent PTXa model^35^.

How Gαi signals to instruct macular HC reversal remained unclear. We hypothesized that Gαi may function downstream of a GPCR for HC reversal. We became interested in GPR156, an orphan class C GPCR with no described function^32–34^. GPR156 is a close homolog of the metabotropic GABA_B_ receptors GABBR1/GABBR2 (Fig. 1b) that are known to signal through Gαi^36–38^. Strikingly, *Gpr156* mouse mutants (*B6N(Cg)-Gpr156*^*tm1.1(KOMP)Vlcg/J*^, hereafter *Gpr156*^*del*^) showed a complete loss of the LPR (Fig. 1c, d), similar to our PTXa model. In controls, we quantified HC orientation in 3 domains across the LPR (Fig. 1a; utricle: LES (lateral extrastriolar), LPR and M (medial) domains; saccule: ANT (anterior), LPR and POST (posterior) domains). In *Gpr156*^*del/del*^ and PTXa mutants, we defined domains of similar size and relative positions using the lateral or anterior edge of the macula as reference in the utricle and saccule, respectively. In *Gpr156*^*del/del*^ and PTXa utricles, HCs in the LES region failed to reverse, HCs in the LPR region lost a bimodal orientation, and HCs in the M region were oriented normally so that all HCs pointed generally laterally (Fig. 1e). Relatedly, in *Gpr156*^*del/del*^ and PTXa saccules, HCs in the ANT region were oriented normally, HCs in the LPR region lost a bimodal orientation, and HCs in the POST region failed to reverse so that all HCs pointed generally anteriorly (Fig. 1f).

Of note, a proportion of PTXa-expressing HCs in the utricle (up to 5% in the LES domain) and the saccule (up to 60% in the POST domain) had an abnormally central basal body, and were thus excluded because their orientation was ambiguous (Supplementary Fig. 1a-d). A similar defect was previously reported in auditory HCs from explants submitted to high doses of purified Pertussis toxin^6^, but absent in auditory HCs expressing PTXa^5,8^. Importantly, the basal body shifted off-center normally in *Gpr156*^*del/del*^ maculae. These results suggest that Gαi, but not GPR156, may have an additional, distinct role in the mechanism ensuring the off-center shift of the basal body itself.

Lastly, we examined HC orientation in the cristae of the vestibular system that normally lack a LPR and where all HCs are uniformly oriented. HC orientation was normal in *Gpr156*^*del/del*^ cristae (Supplementary Fig. 1e). Together these results demonstrate that GPR156 and Gαi are each required to instruct HC reversal and establish the LPR in otolith organs.

### GPR156 is only polarized in *Emx2*^+^ vestibular hair cells

Interestingly, *Gpr156* inactivation precisely recapitulates the *Emx2* mutant phenotype, where loss of the LPR also results from a failure of HCs in the lateral utricle and the posterior saccule to reverse their orientation^27^. *Emx2* expression is restricted to the lateral utricle and the posterior saccule, and absent in cristae^27^ (see Fig. 2e). *Gpr156* could thus be specifically transcribed by EMX2 as an effector in the reversal cascade. We tracked the *LacZ* reporter inserted in the *Gpr156*^*del*^ allele to examine *Gpr156* transcription. Unexpectedly, *Gpr156* was uniformly transcribed across the whole sensory domain in all vestibular organs (Fig. 2a; Supplementary Fig. 2a). In contrast to *Emx2*, a regional transcription factor with broad expression in both HCs and surrounding support cells^27^, *Gpr156* expression was specific to HCs with no organ or regional specificity (Fig. 2b; Supplementary Fig. 2b).

We next used antibodies to detect the GPR156 protein in vestibular organs. Strikingly, we found that GPR156 was asymmetrically enriched at apical HC junctions, but only in the *Emx2*^+^ macular domains. GPR156 planar polarization strictly occurred in HCs sharing the same orientation on one side of the LPR: HCs in the lateral utricle (Fig. 2c, d) and posterior saccule (Supplementary Fig. 2c) (solid arrowheads). In both organs, GPR156 was enriched on the HC side opposite from the basal body. Across the LPR in the medial utricle (Fig. 2c, d) and anterior saccule (Supplementary Fig. 2c), GPR156 was possibly present at junctions at low levels, but not planar polarized (hollow arrowheads). Finally, GPR156 was not planar polarized in crista HCs (Supplementary Fig. 2d) that do not express *Emx2*^27^, do not undergo reversal and lack a LPR. These results indicate that GPR156 planar polarization, rather than *Gpr156* transcription, correlates with spatially restricted *Emx2* expression in HCs (Fig. 2e for summary).

### GPR156 acts downstream of EMX2 and upstream of Gαi

**Fig. 3:**
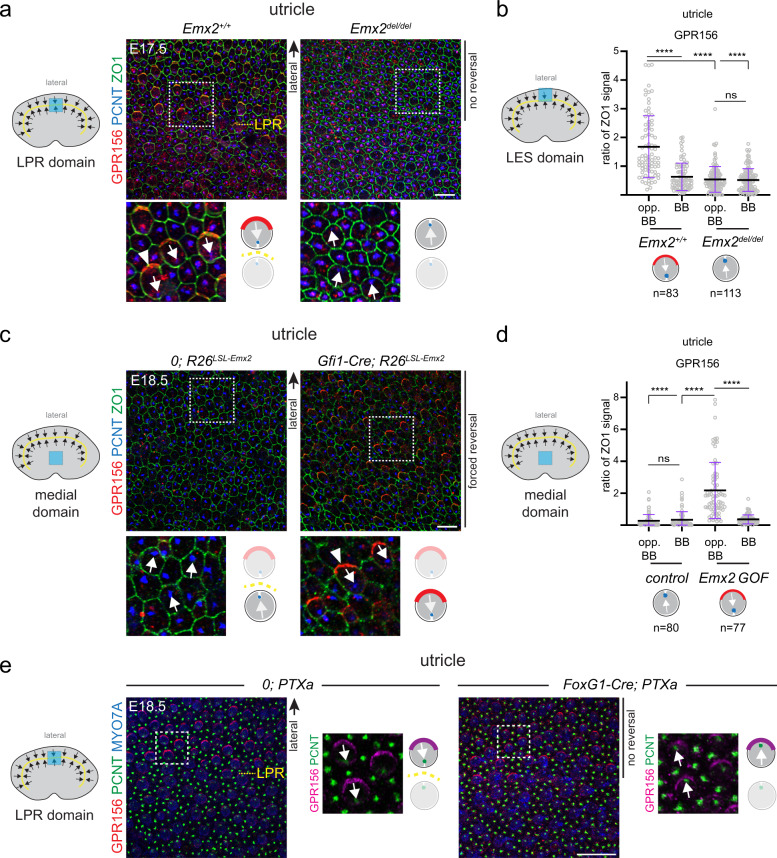
EMX2 > GPR156 > Gαi epistasis in the mouse macular organs. **a** LPR domain in E17.5 utricles. Polarization of GPR156 in lateral HCs (arrowhead) is lost when EMX2 is missing and these HCs fail to reverse. **b** GPR156 enrichment in the lateral utricle (LES) domain. **c** Medial domain in E18.5 utricles. Ectopic expression of *Emx2* reverses HC orientation and induces polarization of GPR156 (arrowhead) in medial HCs. **d** GPR156 enrichment in the utricle medial domain. **e** LPR domain in E18.5 utricles. Polarization of GPR156 in lateral HCs expressing PTXa is intact although these HCs fail to reverse. Utricles are labeled with GPR156, PCNT, and ZO1 (**a**, **c**) or MYO7A (**e**). In **a**, **c**, **e** boxed areas are magnified in the lower panels, and HC orientation and GPR156 distribution are summarized in a cartoon form. In **b**, **d** GPR156 enrichment is measured at the junction opposite (opp. BB) or near (BB) the basal body in the same HC. GPR156 is expressed as ratio of ZO1 signal (mean ± SD; n, HC numbers in 3 or more animals; Kruskal-Wallis test, ****p < 0.0001, ns *p* > 0.9999). Arrows indicate HC orientation based on PCNT-labeled basal body. Utricle schemes indicate the domain imaged or analyzed (blue). Yellow dashed lines represent the LPR in controls. See Supplementary Fig. 3 for related saccule and crista results. Scale bars are 10 µm (**a**, **c**), 20 µm (**e**).

We reasoned that EMX2 may trigger GPR156 planar polarization in HCs, and that when polarized, GPR156 may signal through Gαi to reverse HC orientation. If this general framework is correct, inactivating *Emx2* should abrogate GPR156 polarization in HCs. To test this prediction, we generated a new *Emx2* mutant strain. We found that *in Emx2*^*del/del*^ mutants, the utricle and saccule lacked a LPR as described previously^27,39^, and as observed here upon *Gpr156* or Gαi inactivation (summarized in Fig. 2e). Furthermore, lateral HCs in the *Emx2*^*del/del*^ utricle that failed to undergo reversal lacked GPR156 polarization (Fig. 3a, b). Similarly, posterior HCs in the *Emx2*^*del/del*^ saccule that failed to undergo reversal lacked GPR156 polarization (Supplementary Fig. 3a).

Next, we asked whether ectopic *Emx2* expression in *Emx2*^-^ HCs was sufficient to apically enrich and planar polarize GPR156. We used a mouse strain where *Emx2* is specifically expressed in all HCs upon Cre activation (*Gfi1-Cre; R26*^*LSL-Emx2*^ ^27^). We found that medial HCs in *Gfi1-Cre; R26*^*LSL-Emx2*^ utricles were reversed in orientation as reported previously^27^, and that *Emx2* expression triggered GPR156 enrichment and polarization opposite the basal body (Fig. 3c, d). Similarly, ectopic Emx2 expression in the anterior saccule reversed HC orientation and triggered GPR156 enrichment and polarization opposite the basal body (Supplementary Fig. 3b). Finally, cristae HCs that normally do not express *Emx2*^27^ were reversed in orientation in *Gfi1-Cre; R26*^*LSL-Emx2*^ *mutants* as reported previously^27^, and enriched GPR156 opposite the basal body as well (Supplementary Fig. 3c). Altogether, we conclude that EMX2 is both necessary and sufficient to trigger GPR156 apical enrichment and polarization along with HC reversal in a HC-autonomous manner. To further confirm that EMX2 acts upstream of GPR156, we immunolabeled EMX2 in *Gpr156*^*del/del*^ maculae. We found that similar to controls, EMX2 was still expressed and zonally restricted to the lateral utricle and posterior saccule, in spite of failed HC reversal in these compartments (Supplementary Fig. 3d, e).

Next, we sought to determine whether Gαi acts downstream of GPR156. If so, the junctional polarization of GPR156 should remain intact when Gαi function is inactivated. Because Gαi inactivation prevents HC reversal in the *Emx2* domains (see Fig. 2e), unchanged GPR156 enrichment would be in proximity to the basal body, instead of its normal location opposite the basal body. In the lateral utricle and posterior saccule of *FoxG1-Cre; PTXa* mutants, GPR156 polarized enrichment was indeed intact although HCs failed to reverse orientation, and aberrantly found on the basal body side (Fig. 3e, Supplementary Fig. 3f). These results indicate that normal enrichment of GPR156 cannot direct HC reversal when Gαi signaling is inactivated. In summary, HC reversal is directed by an epistatic EMX2 > GPR156 > Gαi cascade.

### Gpr156 drives hair cell reversal in zebrafish neuromasts

**Fig. 4:**
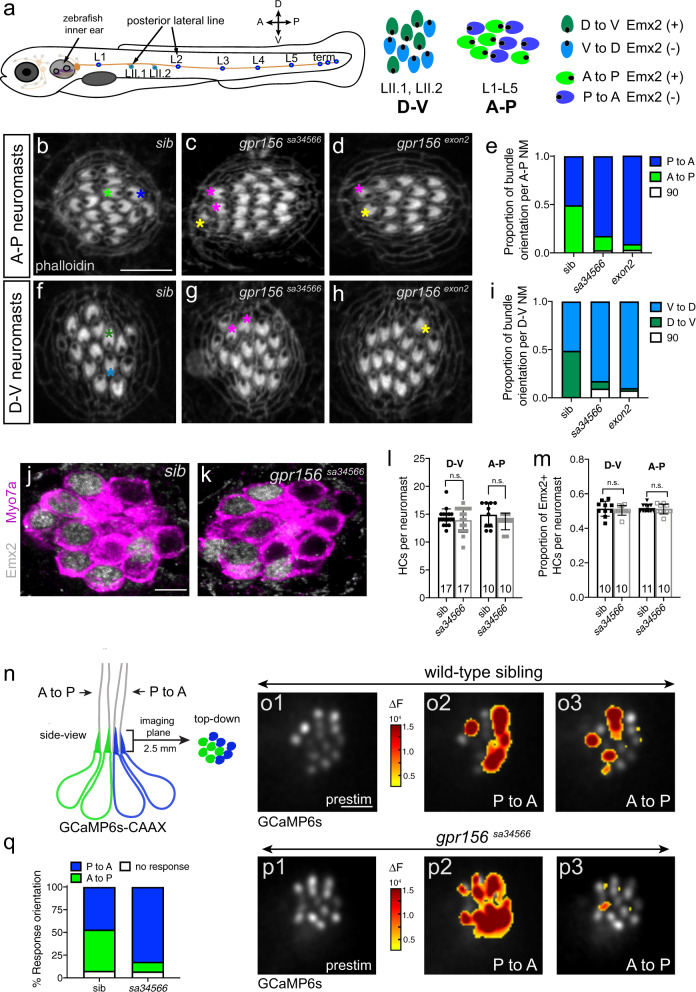
Gpr156 dictates hair cell orientation reversal and directional sensitivity in zebrafish neuromasts. **a** Schematic of the lateral-line system in a 5 day-post-fertilization zebrafish. Neuromast HCs show binary orientation along the antero-posterior (A–P) or dorso-ventral (D–V) axis as depicted. Emx2 is only expressed in HCs of one orientation in each neuromast (green D to V and A to P HCs). HC orientation is indicated by a black dot representing the off-center basal body. **b**–**i** Phalloidin labeling in neuromasts reveals HC orientation by the lack of signal above the off-center basal body. In wild-type siblings (**b**, **e**, **f**, **i**), neuromasts contain an equal proportion of HCs with either orientation. In *gpr156* mutants (**c**–**e**, **g**–**i**), there are more P to A (**c**–**e**) and V to D (**g**–**i**)-oriented HCs compared to wild-type siblings (Tukey’s multiple comparison test, P to A *exon2* allele *p* < 0.0001, sa34566 allele *p* < 0.0001; V to D exon2 allele *p* < 0.0001, *sa34566* allele *p* < 0.0001). Green and blue asterisks highlight the two HC orientations in wild-type sibling neuromasts. Magenta and yellow asterisks highlight outlier HCs oriented 180° or 90° compared to the majority of HCs in *gpr156* mutants. *n* = 10 neuromasts and *N* ≥ 8 animals per genotype, examined at 5 dpf. **j**, **k** Emx2 and Myo7a co-labeling in neuromasts. Wild-type siblings and *gpr156* mutants neuromasts have a similar number of HCs (**l**) (mean ± SEM; unpaired t-test (two-tailed), A-P *p* = 0.1686; Mann–Whitney test (two-tailed), D-V *p* = 0.8547), and a similar proportion of HCs express Emx2 per neuromast (**m**) (mean ± SEM; unpaired t-test (two-tailed), A-P *p* = 0.5756; Mann–Whitney test (two-tailed), D-V *p* = 0.4805). In **l**–**m** the number of neuromasts (n) examined at 5 dpf in *N* ≥ 8 animals per genotype is indicated. **n** Scheme showing the GCaMP6s calcium reporter (blue and green) and the imaging plane in a neuromast. **o1**, **p1** Baseline gray scale GCaMP6s images of the hair bundle imaging plane in wild-type siblings (**o1**) and *gpr156* mutants (**p1**; sa34566 allele). **o2**, **o3**, **p2**, **p3** Spatial patterns of GCaMP6s calcium signal increases in hair bundles during P to A (**o2**, **p2**) or A to P (**o3**, **p3**) directed fluid-jet stimulation. GCaMP6s signals are colorized according to the ∆F heat maps and superimposed onto prestimulus (prestim) baseline images (**o1**, **p1**). **q** In wild-type siblings, GCaMP6s signals are detected during both P to A and A to P directed stimulation (**o2**, **o3**). In contrast, compared to wild-type, in *gpr156* mutants, significantly more hair bundles respond to P to A directed stimulation (**p2**–**p3**) (Sidak’s multiple comparison test, P to A *p* = 0.0008; *n* = 8 neuromasts per genotype and *N* = 4 wild-type and *N* = 3 mutant animals, examined at 5 dpf. See Supplementary Fig. 4 for individual HC responses). NM, neuromast; sib, wild-type sibling. Scale bars are 5 µm (**b**–**d** and **f**–**h**, **j**, **k**, **o1**–**3**, and **p1**–**p3**).

Reminiscent of mouse macular organs, zebrafish neuromasts in the posterior lateral line have two populations of HCs with opposing orientations. Neuromast HCs are oriented to respond to bidirectional fluid-flow either along the dorso-ventral (D-V) or antero-posterior (A-P) body axis (Fig. 4a)^22,40^. *Emx2* is only expressed in one HC population, and is required to reverse its orientation compared to the other population^27,28^. Emx2 reverses anterior HCs in A-P neuromasts and dorsal HCs in D-V neuromasts, allowing them to detect A to P and D to V water flow, respectively, and establishing normal bidirectional sensitivity in each neuromast. We generated a new *gpr156* CRISPR/Cas9 mutant carrying an indel in exon 2 (*gpr156*^*exon2*^*)*, and also obtained a ZIRC line carrying a C-terminal point mutation (*gpr156*^*sa34566*^*)*, both expected to result in a premature stop codon. Using phalloidin labeling to reveal HC orientation, we verified that neuromasts in sibling controls had an equal proportion of HCs of each orientation. In contrast neuromasts from both *gpr156* mutants showed a large excess of P to A (Fig. 4b–e) and V to D (Fig. 4f–i) HCs—the orientations normally adopted by Emx2^-^ HCs. *Gpr156* mutant neuromasts contained normal HC numbers however (Fig. 4j–l), and half the HCs expressed Emx2 as in controls despite loss of orientation reversal (Fig. 4j–k, m). As in the mouse, Gpr156 is thus required downstream of Emx2 in zebrafish neuromasts for orientation reversal in most Emx2^+^ HCs.

Largely unipolar neuromasts in *gpr156* mutants were expected to show severely decreased sensitivity to fluid flow detected by Emx2^+^ HCs, in the A to P direction for A-P neuromasts (Fig. 4n). To test this prediction, we crossed a HC-specific GCaMP6s-CAAX reporter line^41^ to *gpr156*^*a34566*^ mutants to record calcium signals in hair bundles during directional fluid-jet stimulation. Using rapid, in vivo imaging, calcium signals were observed in an equal complement of hair bundles during P to A and A to P stimulation in control siblings (Fig. 4o, q). In contrast, the majority of *gpr156* mutant HCs responded to P to A stimulation (Fig. 4p, q). Despite a dramatic loss of bidirectional response, the amplitude of evoked calcium responses in individual hair bundles was largely normal, and a similar overall proportion of hair bundles per neuromast were responsive in controls and *gpr156* mutants (Supplementary Fig. 4). These results suggest that *gpr156* mutant HCs retain normal mechanotransduction, including Emx2^+^ HCs that fail to reverse orientation. Altogether, GPR156 function is thus conserved across mechanosensory epithelia as divergent as the mouse balance organs and the zebrafish lateral line. By similarity, our zebrafish results suggest that *Gpr156* mutant HCs in the mouse lateral utricle and posterior saccule are likely functional, and only reversed in their directional sensitivity.

### GPR156 or Gαi inactivation misorients auditory hair cells

**Fig. 5:**
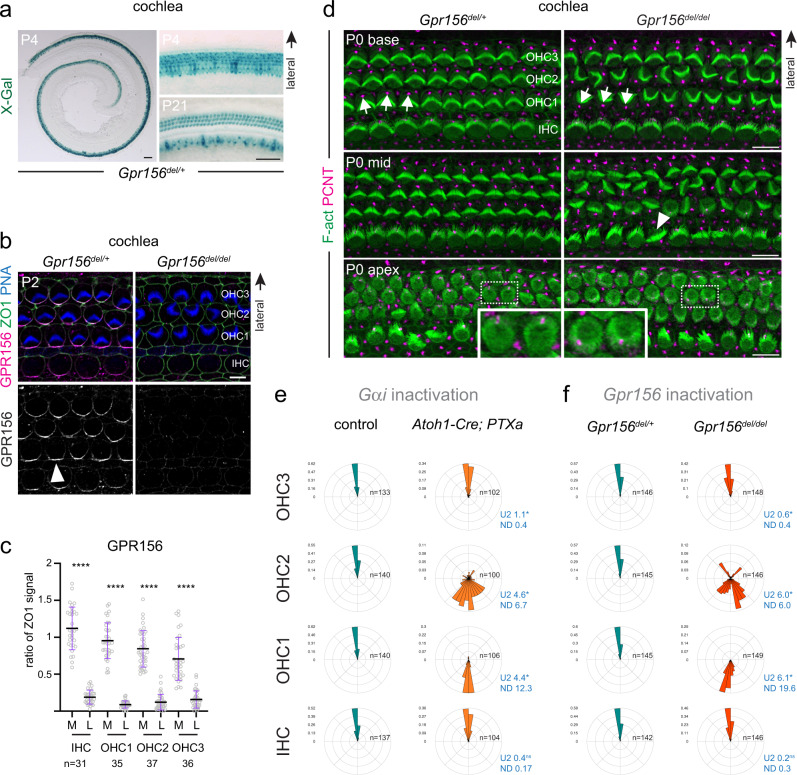
GPR156 expression and hair cell orientation defects upon Gαi or *Gpr156* inactivation in the mouse auditory epithelium. **a** *LacZ* reporter is specifically expressed in HCs in the *Gpr156*^*del/+*^ auditory epithelium at P4 and P21 (right panels: cochlear base). **b** GPR156 immunolabeling shows polarized protein enrichment at the medial HC junction in control (left, arrowhead) but not *Gpr156* mutant cochlear HCs (right). Peanut agglutinin (PNA) labels hair bundles in OHCs. **c** GPR156 enrichment per cochlear HC type at P0 (base) at the medial (M) and lateral (L) junction. GPR156 is expressed as ratio of ZO1 signal (mean ± SD; n, HC numbers in 3 animals; Mann-Whitney test (two-tailed), ****p < 0.0001). **d** P0 auditory epithelium at 3 positions along the cochlea (base, 15%; mid, 50%; apex, 75%). PCNT and phalloidin labeling respectively reveal HC orientation by the position of the off-center basal body and the hair bundle shape. Arrows indicate OHC1 orientation and the arrowhead indicates a rare misoriented IHC. Magnified insets: less mature OHCs at the apex are already reversed in orientation (see also Supplementary Fig. 5c-d). **e**, **f** Circular histograms of P4 HC orientation by row. Histograms show frequency distribution at 50% cochlea position (10° bins in a referential where 90° (top) is lateral and 0° (right) is towards the cochlear base; *n* indicates HC number in 5–7 animals; Watson U2 test of homogeneity; normalized difference (ND) value indicates how many standard deviations separate the circular means of each distribution). In e, *PTXa* indicates the Cre-inducible *R26-LSL-PTXa* allele. Littermate controls for *Atoh1-Cre*; *PTXa* are Cre-negative *PTXa* animals. Scale bars are 100 µm (**a**, left), 50 µm (**a**, right), 5 µm (**b**), 10 µm (**d**).

In contrast to mirror-image HC organization in macular and neuromast organs, in the auditory epithelium a single row of IHC and 3 rows of OHCs adopt a uniformly lateral/abneural orientation (Fig. 1a). Tracking the *LacZ* reporter in the *Gpr156*^*del*^ allele revealed that *Gpr156* was specifically expressed in HCs in the auditory epithelium (Fig. 5a; Supplementary Fig. 5a), as in vestibular organs (Fig. 2a, b). Furthermore, the GPR156 protein was planar polarized medially in all auditory HCs (Fig. 5b, c), and enriched at the apical junction opposite the basal body as in vestibular maculae (Fig. 2c, d; Supplementary Fig. 2c). The GPR156 polarized signal was lost in *Gpr156*^*del/del*^ HCs (Fig. 5b), and was recapitulated with a second antibody against a different GPR156 epitope (Supplementary Fig. 5b). By similarity with the maculae, these results suggest that GPR156 might also influence HC orientation in the auditory epithelium.

We found that *Gpr156* mutants showed a graded range of OHC misorientation across rows: OHC1s were systematically inverted (180°) relative to controls, and OHC2 and OHC3 showed imprecise medial and lateral orientation, respectively (Fig. 5d). In contrast, IHCs were only occasionally misoriented in *Gpr156* mutants (Fig. 5d, arrowhead). Quantification of HC orientation by row in the middle cochlear turn revealed that *Gpr156* inactivation closely mimicked the graded OHC inversion we previously reported upon global inactivation of Gαi function with PTXa (Fig. 5e, f; using *Atoh1-Cre*)^5,8^. Extending quantification to the cochlear base and apex confirmed a similar misorientation profile in *Gpr156* and PTXa mutants (Supplementary Fig. 6a–d). Surprisingly, GPR156-Gαi-mediated HC reversal might thus be integral to uniform auditory HC orientation, and complementary factors may ensure reversal of IHCs and OHC3 (see Discussion).

We also analyzed the auditory epithelium of *Gpr156* mutants at an earlier stage (E17.5) to determine when during development GPR156 alters HC orientation. We assessed multiple positions along the auditory epithelium to capture progressive HC differentiation in time (less mature apex to more mature base^42^; Supplementary Fig. 5c). At the earliest stage cytoskeleton asymmetry could be detected (50% cochlear position from the base), we observed that the kinocilium and basal body were already inverted in *Gpr156*^*del/del*^ OHC1s (Supplementary Fig. 5c). OHC1, OHC2, and OHC3 showed graded HC misorientation outcomes similar to older stages (Supplementary Fig. 5d). We conclude that GPR156 likely regulates the orientation of the early off-center shift of the basal body at pre-hair bundle stages, e.g. intrinsic HC polarization and not subsequent HC reorientation. While GPR156 can reverse auditory HC orientation, only OHC1-2s behave like vestibular or neuromast HCs and fail to reverse along the medio-lateral axis in *Gpr156* mutants.

### EMX2 > GPR156 > Gαi epistasis extends to auditory hair cells

**Fig. 6:**
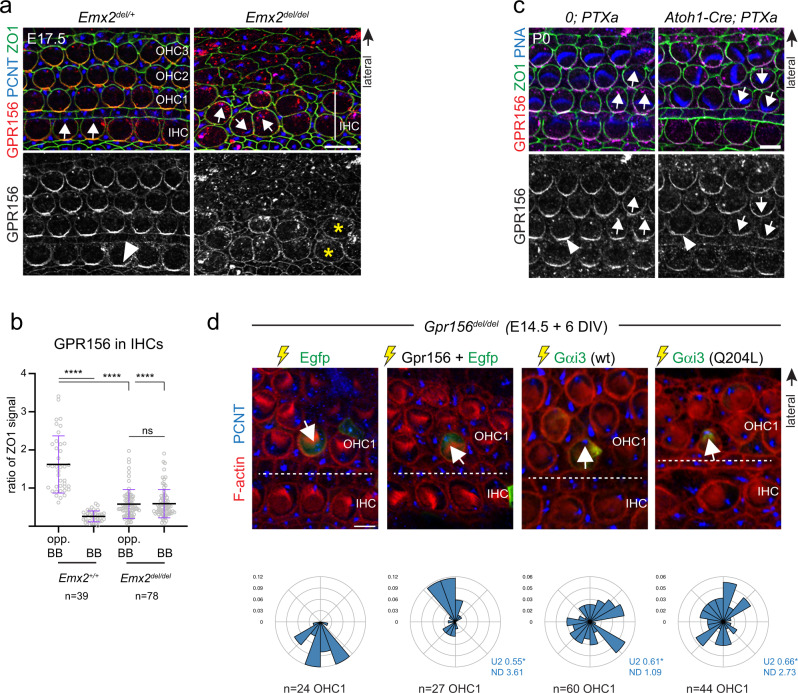
EMX2 > GPR156 > Gαi epistasis and HC-autonomous GPR156 > Gαi function in the mouse auditory organ. **a** E17.5 cochleae labeled with GPR156, ZO1, and PCNT. Polarization of GPR156 in IHCs (arrowhead) is lost in *Emx2* mutants (asterisks), and OHCs are missing (see Supplementary Fig. 6e). **b** GPR156 enrichment in IHCs. GPR156 enrichment is measured at the junction opposite (opp. BB) or near (BB) the basal body labeled with PCNT in the same HC. GPR156 is expressed as ratio of ZO1 signal (mean ± SD; *n*, HC numbers in 3 or more animals; Kruskal-Wallis test, ****p < 0.0001, ns *p* > 0.9999). **c** P0 cochleae labeled with GPR156, ZO1 and peanut agglutinin (PNA). GPR156 is polarized normally (arrowheads) when Gαi is inactivated by PTXa and OHCs show graded inversion by row. **d** Functional rescue of OHC1 orientation in *Gpr156*^*del/del*^ cochlear explants. The constructs indicated were electroporated at E14.5, and the cochleae explanted and cultured for 6 days in vitro (DIV). The orientation of one electroporated OHC1 (green Egfp or Gαi3 signal) is indicated (arrows). Circular histograms show electroporated OHC1 orientation as a frequency distribution for the constructs indicated (20° bins in a referential where 90° (top) is lateral and 0° (right) is towards the cochlear base; n indicates OHC1 number in 10 or more explants representing 2 or more independent experiments; Watson U2 test of homogeneity; normalized difference (ND) value indicates how many standard deviations separate the circular means of each distribution). Note that Egfp and Gpr156 co-electroporation does not guarantee that Egfp^+^ OHC1 express Gpr156, probably explaining why some are not rescued. Arrows indicate HC orientation based on PCNT-labeled basal body (**a**, **d**) or PNA-labeled OHC hair bundle (**c**). In **c** best focus stack slice for PNA signal was combined with lower focus slice for GPR156-ZO1. Scale bars are 10 µm (**a**), 5 µm (**c**, **d**).

Previous work showed that *Emx2* is expressed throughout the auditory epithelium^27^. We examined GPR156 enrichment in *Emx2* mutant cochleae where OHCs were missing, as reported previously (Supplementary Fig. 6e)^39^. IHCs were still present, however, and we observed that medial GPR156 polarization present in control IHCs was lost in *Emx2* mutants (Fig. 6a, b). In contrast, medial GPR156 polarization was maintained in all *Atoh1-Cre; PTXa* auditory HCs in spite of graded OHC inversion across rows (Fig. 6c). These results suggest that in the auditory epithelium as in macular organs, EMX2 is required to planar polarize GPR156. Polarized GPR156 requires Gαi activity to reverse the shift of the basal body.

We next performed functional complementation to confirm that OHC misorientation originated from the loss of GPR156 in HCs. For this analysis we electroporated a Gpr156 construct into E14.5 *Gpr156*^*del/del*^ cochlear explants and cultured for 6 days. We limited analysis to OHC1s where orientation rescue can be unambiguously assessed because OHC1s are systematically and cleanly inverted in *Gpr156* mutants (Fig. 5d, f). Although HC transformation is inefficient and HC morphology suffers in culture, the Gpr156 construct, but not an Egfp control, largely rescued OHC1 inversion (Fig. 6d). Like Gαi^5^, GPR156 thus acts cell-autonomously to define auditory HC orientation.

### GPSM2-Gαi drives morphogenesis, GPR156-Gαi drives reversal

**Fig. 7:**
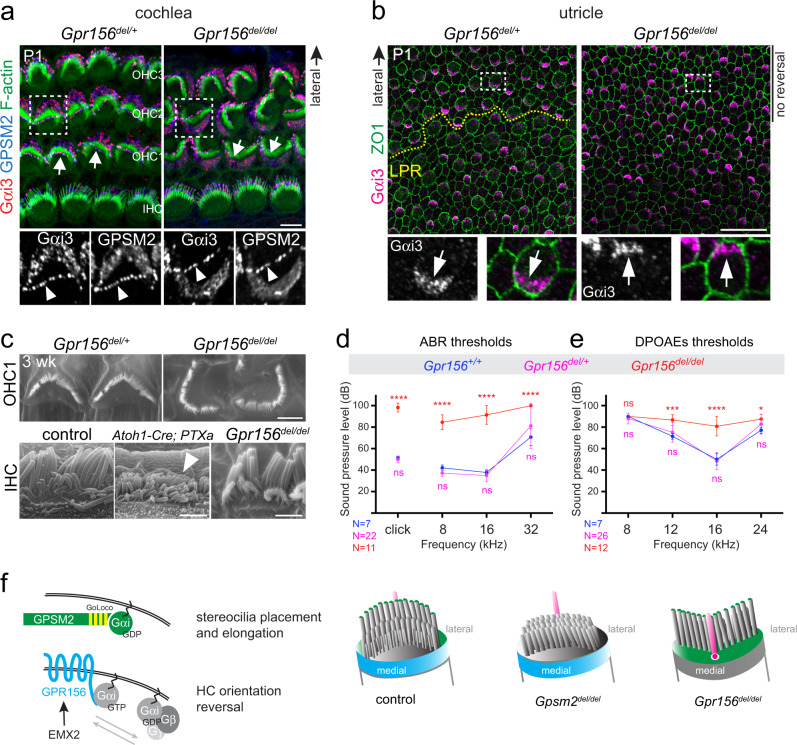
GPR156-Gαi and GPSM2-Gαi are distinct functional modules in developing hair cells. **a**, **b** Normal apical enrichment of GPSM2-Gαi3 (**a**) or Gαi3 (**b**) in P1 *Gpr156* mutant HCs in the cochlea (**a**) and utricle (**b**). Arrows indicate HC orientation. Note how GPSM2-Gαi3 enrichment follows HC orientation when HCs are inverted (OHC1-2s in **a**) or fail to reverse orientation (lateral HCs in **b**). Bottom panels show a magnified view of the HC boxed in the upper panels, and arrowheads point to GPSM2-Gαi3 at stereocilia tips (**a**). See Supplementary Fig. 7a for saccule results. **c** Scanning electron microscopy views of OHC1s (top) and IHCs (bottom) at 3 week (wk). IHC stereocilia stunting is obvious in *PTXa* (arrowhead) but not *Gpr156* mutants. See Supplementary Fig. 7b for larger field views. **d**, **e** Auditory brainstem response (ABR, **d**) and distortion product otoacoustic emissions (DPOAEs, **e**) thresholds at ~4 week of age. X axis indicates broadband (click) and pure tone stimuli for ABR (**d**), or f2 for 2f1-f2 emission for DPOAEs (**e**). Y axis indicates the threshold of sound pressure level eliciting a response (**d**) or generating DPOAEs above noise floor (**e**) (mean ± SD; *N* indicates the number of animals tested per genotype; ABR click: Mann-Whitney test (two-tailed), ****p < 0.0001, ns *p* = 0.4943; ABR pure tones: two-way ANOVA with Sidak’s multiple comparisons, ****p < 0.0001, ns is *p* = 0.6928 (8 kHz), *p* = 0.9167 (16 kHz), *p* = 0.1061 (32 kHz); DPOAE: two-way ANOVA with Sidak’s multiple comparisons, *Gpr156*^*+/+*^ vs *Gpr156*^*del/del*^: ns *p* > 0.9999, ***p = 0.0001, ****p < 0.0001; *p = 0.0138; *Gpr156*^*+/+*^ vs *Gpr156*^*del/+*^: ns is *p* = 0.9103 (8 KHz), *p* = 0.6116 (12 kHz), *p* = 0.9512 (16 kHz), *p* = 0.299 (24 kHz)). **f** Working model. GPSM2-Gαi(GDP) (green) instructs stereocilia placement and elongation. In contrast, GPR156-Gαi(GTP) (blue) triggers HC orientation reversal downstream of EMX2. Scale bars are 5 µm (**a**), 20 µm (**b**), 2 µm (**c**).

We and others previously showed that Gαi is critical for hair bundle morphogenesis in the mouse inner ear^5–11^. In this role, Gαi binds to the GPSM2 scaffold to form a complex that occupies the bare zone and the tips of the abutting tallest stereocilia in HCs (Fig. 1a; see also Fig. 7f). Interestingly however, *Gpsm2* mutants do not show the OHC misorientation^5–8^ observed when Gαi is inactivated by PTXa^5,8^ or when GPR156 is missing (Fig. 5d, f). Gαi might thus work with GPSM2 to instruct hair bundle morphogenesis, and with GPR156 to reverse HC orientation.

If these represent distinct, independent Gαi pathways, the polarized enrichment and function of the GPSM2-Gαi complex should be intact in *Gpr156* mutant HCs. Verifying this prediction, Gαi and GPSM2 were normally co-enriched at the bare zone and at the tips of the tallest stereocilia in auditory HCs in *Gpr156* mutants (Fig. 7a). GPSM2-Gαi was in register with the apical cytoskeleton, even in misoriented OHCs (Fig. 7a). Similarly, in the lateral utricle (Fig. 7b) and the posterior saccule (Supplementary Fig. 7a), Gαi3 crescents were intact but inverted in orientation in *Gpr156* mutants, in register with inverted HCs caused by failed reversal in these domains (Fig. 1c–f). In support of normal GPSM2-Gαi HC function, hair bundle morphology appeared normal in *Gpr156*^*del/del*^ auditory HCs. In stark contrast, *Gpsm2* or Gαi inactivation results in dysmorphic hair bundles^8–11^. For example, IHC stereocilia were severely stunted in PTXa mutants but elongated to normal heights in *Gpr156* mutants (Fig. 7c).

### Mature organs in *Gpr156* mutants

To ask whether GPR156-Gαi has another role besides regulating HC orientation, we next analyzed *Gpr156* mutants after inner ear development is completed. In the cochlea, graded OHC inversion persisted without causing HC death at 3 weeks of age (Supplementary Fig. 7b). To assess the impact of normally shaped yet misoriented hair bundles, we recorded auditory brainstem responses (ABR) in *Gpr156* mutants. ABR revealed significant hearing loss with broadband (click) and pure tone (8, 16, 32 kHz) stimuli (Fig. 7d). In addition, we observed impaired distortion product otoacoustic emissions (DPOAEs), which indicates a specific defect in OHC function (Fig. 7e). OHC misorientation thus likely contributes to hearing loss in absence of GPR156. HC misorientation (e.g. lack of HC reversal) persisted as well in mature *Gpr156* mutant utricles (Supplementary Fig. 7c). In contrast, macular zoning appeared normal in 4 week-old *Gpr156* mutants, with a distinct striolar region (Supplementary Fig. 7d). Type I and II HCs were distributed normally in the lateral utricle where HCs failed to reverse (Supplementary Fig. 7e, f). Finally, HC density by domain and total macular surface area was also unchanged in mature *Gpr156* mutant utricles (Supplementary Fig. 7g).

In summary, GPR156 specifically modulates Gαi activity to reverse HC orientation, and does not obviously influence another patterning process in sensory epithelia. Gαi is thus critical for two distinct aspects of HC development at least—HC orientation (with GPR156) and hair bundle morphogenesis (with GPSM2). As PTXa prevents both Gαi roles, PTXa recapitulates both *Gpr156* and *Gpsm2* phenotypes that are otherwise non-overlapping (Fig. 7f).

### GPR156 likely drives hair cell reversal via G proteins

GPSM2 is known to specifically bind, sequester, and accumulate Gαi in its GDP-bound form, forming an unconventional yet highly conserved polarity complex^13,14,43^. In HCs, GPSM2-Gαi(GDP) first forms a planar polarized crescent at the apical membrane on the basal body side, the bare zone in auditory HCs (Fig. 1a)^5^. We hypothesized that, by contrast, Gαi might act as a conventional switch downstream of GPR156 acting as a classic GPCR to define HC orientation. We were unable to reliably detect Gαi along with GPR156 at the medial HC junction using immunolabeling, possibly because low Gαi amounts are sufficient for GDP > GTP guanine nucleotide exchange and signaling. To gather alternative evidence, we first performed co-immunoprecipitation experiments in HEK293 cell extracts. We were able to pull down Gαi3 with 2HA-GPR156, but not HA (Supplementary Fig. 7h). Reciprocally, 2HA-GPR156 was pulled down upon Gαi3, but not empty vector transfection (Supplementary Fig. 7i). Second, we electroporated a wild-type or constitutively active (Q204L) Gαi3 construct in *Gpr156*^*del/del*^ cochlear explants. Strikingly, both constructs conferred significant rescue of OHC1 orientation from medial to lateral, although not as efficiently as Gpr156 itself (Fig. 6d).

In summary, 4 independent lines of evidence suggest that Gαi does signal downstream of GPR156 at the HC junction. **1**) *Genetic: Gpr156* and PTXa mutants share the same cochlear and macular misorientation phenotypes (Figs. 1 and 5), with the expected epistasis in both systems (Figs. 3 and 6a–c). Notably, normal polarization of GPR156 cannot orient HCs properly if Gαi is inactivated (Figs. 3e and 6c; Supplementary Fig. 3f). **2)** *Phylogenic*: The closest homologs of GPR156, the GABBR1/GABBR2 receptors (Fig. 1b), signal through Gαi^36–38^. **3)** *Physical:* GPR156 and Gαi3 interact (Supplementary Fig. 7h-i). **4)** *Functional:* overexpressing Gαi3 can promote HC reversal when GPR156 is absent (Fig. 6d).

### GPR156 polarized enrichment relies on core PCP patterning

Our results indicate that *Gpr156* is specifically expressed in HCs and that GPR156 is enriched at the medial HC junction in the auditory epithelium (Fig. 5a–c). GPR156 localization suggests that the GPR156 > Gαi module could implement HC reversal in collaboration with factors that regulate cytoskeleton asymmetry in single HCs, or with global signals that regulate HC orientation at cell–cell junctions (PCP proteins)^2^. To start investigating this question, we analyzed GPR156 in relation to other polarity proteins at the HC junction.

We found that GPR156 distribution overlapped with both aPKC and PARD6B, two polarity proteins of still unknown function at the medial HC junction (Supplementary Fig. 8a, b)^5,6^. GPR156 extended further basally down the junction compared to aPKC and PARD6B. We observed a reduced enrichment of aPKC-PARD6B in *Gpr156* mutant HCs. Despite reduced amounts, aPKC-PARD6B remained enriched opposite the basal body even when OHCs were misoriented (Supplementary Fig. 8c). aPKC and PARD6B are thus possible GPR156 partners at the medial HC junction.

Next, we monitored PARD3A^5,44^ and DAPLE^45^, two polarity proteins that occupy the lateral HC junction opposite from GPR156/aPKC/PARD6B. At E16.5, X-gal staining revealed a decreasing gradient of *Gpr156* expression along the cochlea from the more mature base to the less differentiated apex (Supplementary Fig. 8d). At mid position, only traces of GPR156 were detected at the medial junction whereas PARD3A/DAPLE enrichment was already evident at the lateral junction (Supplementary Fig. 8e, f). We conclude that GPR156 is enriched independently from, and later than, PARD3 and DAPLE. Accordingly, PARD3A and DAPLE were unchanged in their distribution in *Gpr156* mutant HCs, again following HC misorientation and remaining enriched on the basal body side (Supplementary Fig. 8g, h). Surprisingly however, GPR156 remained strictly enriched at the medial HC junction in *Daple* mutants in spite of altered HC orientation and a severely disrupted apical cytoskeleton^45^ (Supplementary Fig. 8i). In summary, opposing enrichment of aPKC/PARD6B and PARD3A/DAPLE is in register with HC-intrinsic cues and defined by HC orientation in *Gpr156* mutants. In stark contrast, junctional enrichment of GPR156 is not defined by HC orientation, suggesting that GPR156 polarization depends on cues external to HCs, like core PCP proteins^17,18^.

**Fig. 8:**
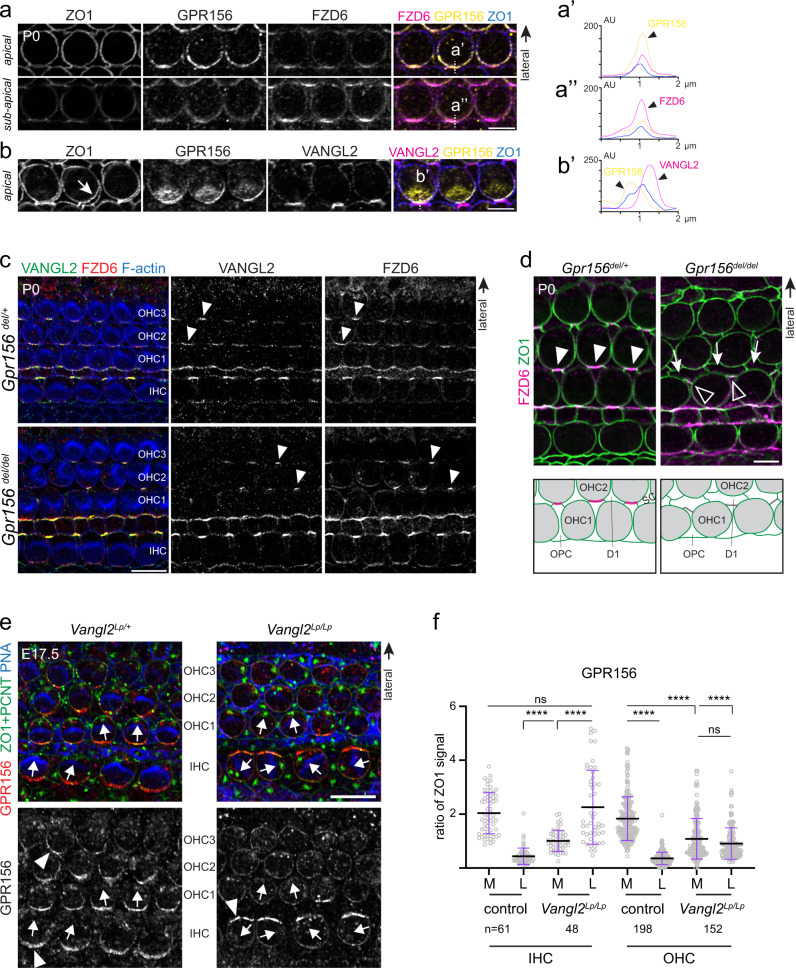
Relationship between GPR156 and core PCP factors at the medial HC junction in the cochlea. **a**, **b** P0 wild-type OHC2s. GPR156 co-labeling with ZO1 and either FZD6 (**a**) or VANGL2 (**b**). **a** GPR156 and FZD6 overlap at the medial junction, but the bulk of GPR156 is planar polarized at ZO1 level (top panels; apical), above the bulk of FZD6 (bottom panels; sub-apical, 0.4 µm more basally in the same confocal stack). **b** VANGL2 is mostly enriched on the support cell side of the medial HC junction, as apparent when TCA fixation separates the OHC and support cell plasma membranes (arrow). a′, a″, and b′ show a plot profile of signal intensity at the medial HC junction along the line shown in the merge panels (GPR156, yellow; ZO1, blue; FZD6 or VANGL2, magenta). **c** VANGL2 and FZD6 co-labeling in P0 *Gpr156*^*del*^ cochleae. Co-enrichment at medial OHC junctions (arrowheads) is still observed in *Gpr156* mutants, but less consistently. **d** FZD6 and ZO1 co-labeling in P0 *Gpr156*^*del*^ cochleae. Reduced FZD6 enrichment in *Gpr156* mutants (hollow arrowheads) compared to controls (solid arrowheads) corresponds to regions with aberrant support cell-support cell contacts. Arrows show support cells with an abnormal apical domain shape and location (schematized below each panel). Support cells: OPC, outer pillar cell, D1, Deiter 1 cell. **e** GPR156 labeling in E17.5 *Vangl2*^*Lp*^ cochleae. In *Vangl2*^*Lp/Lp*^ homozygotes, polarized GPR156 enrichment at the junction is lost in OHCs, but inverted in IHCs (arrowheads). Arrows show HC orientation based on the position of the basal body (PCNT) and the shape of the hair bundle (peanut agglutinin, PNA). **f** GPR156 enrichment at the medial (M) and lateral (L) junction in the same HC. GPR156 is expressed as the ratio of ZO1 signal (mean ± SD; *n*, HC numbers in 3 or more animals; Kruskal-Wallis test, ****p < 0.0001, ns is *p* > 0.9999 (IHC), *p* = 0.8111 (OHC)). Controls are pooled *Vangl2*^*+/+*^ and *Vangl2*^*Lp/+*^ samples. Scale bars are 5 µm (**a**, **b**, **d**), 10 µm (**c**, **e**).

To explore this possibility, we compared distribution of GPR156 with two core PCP proteins enriched at the medial HC junction that interact together through their extracellular domains: FZD6 originating from the HC^46^ and VANGL2 originating from the adjacent support cell^47^. We co-immunolabeled GPR156 and FZD6 or VANGL2 using ZO1 as an independent apical junction marker. GPR156 and FZD6 distributions partially overlapped but GPR156 was largely confined to ZO1-labeled apical junctions, and the bulk of the FZD6 signal was located more basally (Fig. 8a). Taking advantage of occasional junction disruption during TCA fixation, we observed that GPR156 and VANGL2 were closely juxtaposed but respectively localized to the medial HC and lateral support cell junction, as expected (Fig. 8b)^47^.

To determine whether GPR156 functionally interacts with the PCP machinery, we first examined the distribution of FZD6 and VANGL2 in *Gpr156* mutants. We observed occasional loss of FZD6 and VANGL2 co-enrichment, yet many instances of normal enrichment at the medial HC junction (Fig. 8c, arrowheads). Closer examination using ZO1 to label all junctions revealed that lost or reduced FZD6-VANGL2 enrichment occurred at abnormal contacts between two support cells where one of the support cell should have instead contacted an OHC (Fig. 8d). GPR156 absence thus disrupts the HC-support cell checkerboard, perhaps because early OHC misorientation (Supplementary Fig. 5c, d) interferes with the apical intercalation of the phalangeal processes from support cells. This disruption of cellular architecture in the auditory epithelium likely contributes to auditory dysfunction (Fig. 7d, e) along with OHC misorientation (Fig. 5d, f) in *Gpr156* mutants.

Finally, we asked whether disrupting core PCP signaling affects GPR156 distribution. Core PCP proteins are largely co-dependent for their asymmetric junctional enrichment^46,48,49^. We used the *Vangl2 Looptail* (*Lp*) mutant where the FZD6-VANGL2 complex at the medial HC junction is missing^46^. Interestingly, GPR156 enrichment was severely reduced and unpolarized in *Vangl2*^*Lp/Lp*^ OHCs (Fig. 8e, f). In contrast, GPR156 asymmetric enrichment appeared to be retained but switched to the lateral junction in *Vangl2*^*Lp/Lp*^ IHCs (Fig. 8e, f). We conclude that GPR156 relies on core PCP patterning for asymmetric enrichment in OHCs and for medial enrichment in IHCs. Although definitive conclusions await results in other core PCP mutants, EMX2 > GPR156 > Gαi appears to be an optional module that reverses HC orientation along an axis defined by core PCP proteins.

## Discussion

Previous work investigated and ruled out the class C GPCR GPR156 as a potential metabotropic GABA receptor, leaving its function unknown^32,34^. We now define GPR156 as a highly conserved cell polarity determinant. In contrast to GPR156, GPCRs previously involved in planar polarity belong to the adhesion (B2) class (CELSR1-3) or the Frizzled (F) class (FZD3, 6). In mouse otolith organs and zebrafish neuromasts, polarized GPR156 intrinsically triggers orientation reversal in *Emx2*^+^ HCs to generate binary HC orientations. Our results also show that GPR156 requires Gαi as effector for reversal. As mutant HCs appear reversed as early as they break apical symmetry (Supplementary Fig. 5c, d), GPR156-Gαi signaling likely acts to reverse the orientation of the basal body when its shifts off-center before the hair bundle is established. A proportion of vestibular, but not auditory HCs expressing PTXa maintained an abnormally central basal body (Supplementary Fig. 1). Gαi may thus also act independently from GPR156 to allow the off-center migration itself, as proposed previously using purified Pertussis toxin^6^. Significantly, inactivating the EMX2-GPR156-Gαi module does not disrupt the axis along which macular or neuromast HCs are oriented, but only the direction of HC orientation along that axis. It thus appears that GPR156-Gαi reads core PCP patterning and reverses the orientation of the basal body shift otherwise solely determined by core PCP proteins^50^. This notion is supported by partial colocalization of GPR156 and the PCP protein FZD6, and by the loss or reversal of GPR156 polarization when PCP function is disrupted (Fig. 8). GPR156 is probably not a core PCP protein as it is not directly required for FZD6-VANGL2 enrichment (Fig. 8).

As a transcription factor, EMX2 was expected to bind a regulatory region of a target gene(s) that would be transcribed and implement HC reversal regionally. Instead, we show that EMX2 is necessary and sufficient to polarize the protein distribution of a receptor uniformly transcribed in all HCs. It remains possible that EMX2 activates a still unknown gene(s) whose product regionally promotes GPR156 polarized trafficking or junctional enrichment. After GPR156-Gαi is polarized at the HC membrane, it also remains unclear how signaling is initiated. GPR156 could rely on an agonist, either a secreted protein or the extracellular domain of a protein in neighboring support cells, similar to intercellular communication among core PCP proteins^2,17,18^.

Another intriguing question is whether the *Gpr156* mutant could be used to understand the functional significance of bimodal HC orientation in otolith organs. Unlike *Emx2*^27^ and Gαi, *Gpr156* expression is limited to HCs, and the role of GPR156 could be limited to HC orientation reversal. This is supported by a lack of other obvious patterning defects in adult *Gpr156* mutants. Furthermore, HCs that fail to reverse in *gpr156* mutant neuromasts show normal mechanotransduction. *Gpr156* mutants thus represent an exciting opportunity to investigate how HC reversal affects afferent organization and physiology as well as balance behavior in adult animals. Work to address these long-standing questions is underway.

The mouse auditory epithelium lacks mirror-image HC organization, but nevertheless surprisingly depends on GPR156-Gαi for proper HC orientation. Importantly, *Gpr156* mutants share the graded OHC inversion we first reported upon Pertussis toxin expression^5,8^, and thus validate this phenotype as biologically relevant. Why would orientation reversal be needed for auditory HCs to be uniformly oriented? GPR156-Gαi function in the cochlea may be an evolutionary carry-over from the lateral utricle and posterior saccule regions that express *Emx2*. The entire auditory epithelium is indeed part of the *Emx2* lineage, unlike the medial utricle, anterior saccule, and the cristae^27^. Interestingly, mirror-image HC organization is a classic feature of the auditory papilla in lizards^51,52^ which is absent in the auditory epithelium of birds or mammals. Opposing IHC-OHC1 orientation observed in *Gpr156* and PTXa mutants is particularly reminiscent of the opposing orientations of low- and high-frequency auditory HCs in the lizard papilla^52^. It is thus possible that the recruitment of GPR156-Gαi introduced HC reversal in fish neuromasts and otolith organs, and that concomitant reversal of high-frequency HCs in the auditory papilla abolished mirror-image organization in the cochlea of modern amniotes.

Remarkably, inactivating GPR156-Gαi fully abrogates reversal in *Emx2*^*+*^ HC in the maculae and neuromasts, whereas the cochlear phenotype appears incomplete. OHC1 and OHC2 are robustly inverted, whereas IHC and OHC3 are much less affected (Fig. 5). Interestingly, previous studies reported consistent inversion of the complementary HC types: IHC in *Fzd3,6*^46^ and OHC3 in *Vangl2*^53,54^ mutants. At the time, these results were somewhat surprising because core PCP proteins are generally thought to pattern epithelia locally, with complete loss of function resulting in randomized, and not inverted, cell orientation^17,18^. It is thus possible that all cochlear HC types normally undergo reversal to switch their orientation from medial/neural to lateral/abneural by using distinct polarity complexes.

Adult *Gpr156* mutant cochleae interestingly retain misoriented OHCs without evidence of dysmorphic hair bundles or HC death. OHC misorientation might thus be one contributing factor to severe hearing loss observed in these mutants. We indeed show reduced distortion products sounds (DPOAEs) that are generated by OHCs, and HC mechanotransduction appears normal in absence of Gpr156 in neuromasts HCs. A confounding factor, however, is that OHC misorientation in *Gpr156* mutants also affects the apical morphology of neighboring support cells (Fig. 8d). In turn, this is likely to affect cochlear mechanics. Related apical morphology defects in support cells were actually proposed to cause hearing loss in conditional *Vangl2* mutants where, by contrast, OHC3 misorientation is largely corrected postnatally^54^. Finally, we cannot exclude that GPR156 plays another role in HCs and affects their physiology, potentially also contributing to hearing loss.

In cell polarity contexts, notably during orientation of the mitotic spindle^15,16^, polarized Gαi is widely reported as dissociated from Gβγ and sequestered and enriched in its GDP form when binding the GoLoco domains of GPSM2^13,14,43^. In HCs, Gαi and GPSM2 are colocalized and co-dependent at the bare zone and stereocilia tips (Fig. 7f), and we thus proposed that Gαi plays a similar unconventional role in these HC compartments^5^. Here we extend this work by uncovering a parallel role for Gαi during apical HC differentiation (Fig. 7f). We propose that GPR156 acts as a conventional GPCR with Guanine nucleotide Exchange Factor (GEF) activity to activate the heterotrimeric Gαiβγ complex and direct HC reversal. HC reversal is probably signaled by Gαi-GTP, as inhibiting Gβγ pharmacologically did not invert OHC orientation in vitro^55^. Remarkably, *Gpsm2* and *Gpr156* mutants have no phenotype in common (Fig. 7f). In contrast, our PTXa model exhibits compound phenotypes: (1) disorganized and stunted stereocilia as in *Gpsm2* and *G*α*i* mutants^8–11^, and (2) failed HC orientation reversal as in *Gpr156* and *Emx2*^27,39^ mutants. High amounts of GPSM2-sequestered Gαi(GDP) vs low amounts of Gαi cycling between its Gαi(GDP)βγ and Gαi(GTP) conformations could explain why Gαi is only reliably immunodetected with GPSM2.

We previously reported that another GEF, the non-receptor DAPLE, is enriched at the lateral HC junction along with the core PCP protein DVL2, adjacent to GPSM2-Gαi at the lateral bare zone. DAPLE can bind both Gαi and DVL2^56^, and *Daple* mutants show both HC misorientation and dysmorphic hair bundles^45^. We thus proposed that DAPLE links GPSM2-Gαi to core PCP proteins to couple the asymmetric HC cytoskeleton to the machinery defining HC orientation. Here, the rationale for complementary lateral (DAPLE) and medial (GPR156) GEF activity at the HC junction remains unclear. Indeed, we found that GPR156 does not rely on DAPLE for its medial enrichment, whereas DAPLE remained polarized in register with the cytoskeleton in misoriented *Gpr156* mutant OHCs. Of note, DAPLE might influence HC polarity independently from Gαi as it has other functional domains besides GEF^56^.

In summary, Gαi is emerging as a critical regulator of directional sensitivity in mechanosensory epithelia. Gαi confers graded stereocilia height and thus a directional response to individual hair bundles with GPSM2^8–11^. Gαi also reverses HC (and thus hair bundle) orientation with GPR156 to create a mirror-image organization in otolith organs and fish neuromasts.

## Methods

### Mice

The *Gpr156*^*del*^ strain (*B6N(Cg)-Gpr156*^*tm1.1(KOMP)Vlcg/J*^; MGI:5608696) was produced by the Knockout Mouse Project (KOMP). The following published strains were used: *Daple*^*del*^ (C57BL/6N-Ccdc88c^tm1.1(KOMP)Mbp^; MGI:5548562), *R26*^*LSL-mycPTXa*^ (*Gt(ROSA)26Sor*^*em1(ptxA)Btar*^; MGI:6163665)^8^. *R26*^*LSL-Emx2*^ (*Rosa*^*Emx2-Egfp*^)^27^ was produced by Doris Wu at NIH/NIDCD. *Vangl2*^*Lp*^ is (LPT/LeJ; MGI:1857642). References for the Cre lines are as follows: *Atoh1-Cre* (*Tg(Atoh1-cre)1Bfri*, MGI:3775845)^57^, *FoxG1-Cre (Foxg1*^*tm1(cre)Skm*^; MGI:1932522)^58^, *Gfi1-Cre* (*Gfi1*^*tm1(cre)Gan*^; MGI:4430834)^59^. The *Emx2*^*del*^ strain was generated in the C57BL/6J background with CRISPR/Cas9 to entirely delete the coding portion of *Emx2* first exon. The following guide RNAs were used: 5′- TCGGCGCAGCATGTTTCAGC-3′ and 5′- AGTTTCAGAACCAAGAACCC-3′. A founder mouse that contained the expected ~500 bp deletion was identified by standard PCR. The strain was used for analysis after breeding with wild-type C57BL/6J animals for 2 generations to avoid potential unwanted genomic alterations. Wild-type animals were either C57BL/6J inbred, or C57BL/6J x FVB/NJ outbred. Primers used for genotyping are indicated in Supplementary Table 1. Animals were maintained under standard housing with a 14 hour light/10 hour dark cycle, ambient temperature and normal humidity. All animal work was reviewed for compliance and approved by the Animal Care and Use Committee of The Jackson Laboratory (Animal Use Summary #14012).

### Mouse cochlear culture and electroporation

Inner ears from *Gpr156*^*del/del*^ mutant mice were collected at E14.5 in HBSS + Hepes. Circular plasmid DNA mixed with fast green was injected in the cochlear duct at 2 µg/µl (caggs-Egfp, caggs-Gpr156 (Q6PCP7-1), caggs-Gαi3 (wt); caggs-Gαi3 (Q204L); mouse coding sequences). The whole inner ear was then electroporated (27 V, 27 ms, 6 square pulses at 950 ms intervals; BTX ECM 830), and the membranous labyrinth was dissected away from the condensed mesenchyme and embedded in a 8 µl drop of Matrigel (50% in DMEM; Corning 356237). Explants were cultured for 6 days in Dulbecco’s Modified Eagle Medium (DMEM) with 10% fetal bovine serum and 10 µg/ml ciprofloxacin. Note that co-electroporation of caggs-Egfp and caggs-Gpr156 does not guarantee that all Egfp^+^ OHC1 actually express Gpr156, which can explain why some Egfp^+^ OHC1 are not rescued.

### Mouse immunolabeling

Temporal bones were isolated and either (1) immediately microdissected to expose the cochlear or vestibular epithelia at late fetal or neonate stages, or (2) the cochlea was punctured at the apex to facilitate fixative access for samples past postnatal day (P) 7. Samples were then fixed in paraformaldehyde (PFA 4%; 1 h to overnight at 4 °C) or trichloroacetic acid (TCA 10%; 10 min on ice) depending on antibodies. After PBS rinses, the tectorial membrane was removed (1), or the temporal bone was treated with 0.11 M EDTA overnight at room temperature for decalcification before dissection (2). Dissected samples were permeabilized and blocked in PBS with 0.5% Triton-X100 and bovine serum albumin (1%) for at least 1 h at room temperature before application of the primary antibodies. Both primary and secondary antibodies were incubated overnight at 4 °C, conjugated phalloidin was added with secondaries, and washes were done with PBS + 0.05% Triton-X100. Samples were then post-fixed in PFA 4% for 1 h at room temperature, rinsed, and generally mounted flat between a glass slide (Denville M1021) and a 18 × 18 mm #1.5 coverglass (VWR 48366-045) with Mowiol as mounting medium (Calbiochem/MilliporeSigma 475904). Mowiol (10% w/v) was prepared in 25%(w/v) glycerol and 0.1 M Tris-Cl pH8.5.

Primary antibodies used were: goat anti-GPR156 (Santa Cruz; sc102572; TCA; 1:100), rabbit anti-GPR156 (Novus; NBP1-83402; TCA; 1:100), mouse anti-acetylated Tubulin (Santa Cruz; 23950; PFA; 1:500), rabbit anti-pericentrin/PCNT (Biolegend; PRB-432C; PFA; 1:400), mouse anti-βII-Spectrin/SPTBN2 (BD Transduction Lab; 612562, PFA; 1:200), rat anti-ZO1 (Developmental Studies Hybridoma Bank; R26.4C; TCA; 1:200), rabbit anti-Gαi3 (Santa Cruz; sc-262; PFA; 1:400), chicken anti-Gαi3 (Sigma; GW22489; PFA; 1:400; used for cochlear explants), rabbit anti-DAPLE/CCDC88C (Proteintech; 25769-1-AP; TCA; 1:400), mouse anti-MYO7A (Developmental Studies Hybridoma Bank; 138-1; PFA; 1:500), goat anti-FZD6 (R&D Systems; AF1526; PFA; 1:200), rabbit anti-VANGL2 (gift from Philippe Gros, McGill University; PFA; 1:500), goat anti-GPSM2/LGN (ThermoFisher Scientific; PA5-18646; PFA; 1:200), rabbit anti-βGalactosidase (Cappel discontinued aliquot; PFA; 1:1000; now MP Biomedical 55976), mouse anti-aPKC/PRKCZ (Santa Cruz; sc-216; PFA; 1:100), rabbit anti-PARD6B (Santa Cruz; sc-67393; PFA; 1:100), rabbit anti-PARD3A (Proteintech; 11085-1-AP; PFA; 1:200), rabbit anti-CALB1 (Cedarlane/Millipore; AB1778(CH); PFA; 1:500), goat anti-SPP1/Osteopontin (R&D Systems; AF808; PFA; 1:100), goat anti-SOX2 (Santa Cruz; 17320; PFA; 1:500), rabbit anti-EMX2 (Trans Genic; KO609; PFA; 1:250). Secondary antibodies were raised in donkey or goat and coupled to Alexa Fluor (AF) 488 (used 1:1000), 555 (used 1:1000) or 647 (used 1:500) (ThermoFisher Scientific). Fluorescent conjugated phalloidins were used to reveal F-actin (ThermoFisher Scientific: AF488, A12379; AF555, A34055. Biotium: CF405M, 89138-126).

### Mouse anatomical and molecular analyzes using light microscopy

Controls for *Gpr156*^*del/del*^ were *Gpr156*^*del/+*^ littermates, as heterozygotes did not show physiological, anatomical or molecular defects compared to wild-type littermates. This allowed to reduce the number of animal produced for this study by mostly breeding *Gpr156*^*del/del*^ with *Gpr156*^*del/+*^ for tissue collection. Controls for *Emx2*^*del/del*^ and *Vangl2*^*Lp/Lp*^ were either wild-type or heterozygote littermates as a single mutant gene copy did not obviously affect the phenotype investigated. Controls for Pertussis expression (*FoxG1-Cre; R26*^*LSL-PTXa*^ or *Atoh1-Cre; R26*^*LSL-PTX*^) or *Emx2* ectopic expression (*Gfi1-Cre; R26*^*LSL-Emx2*^) were Cre-negative littermates also carrying a heterozygote *R26* knock-in insertion. Both sexes are represented in all anatomical and molecular studies but data is not presented by sex since there is no evidence that developmental mechanisms defining hair cell orientation differ in males and females.

Most images were acquired on a LSM800 line scanning confocal microscope using the Zen2.3 or Zen 2.6 softwares, regular confocal capture with an Airyscan detector and a 63×/1.4 NA oil objective lens (Carl Zeiss AG). Brightfield signals were acquired on a Leica DM5500B. Images were processed with Adobe Photoshop (CS6 or CC 2020) with the same treatment applied to different genotypes or conditions in the same experiment.

For quantification of hair cell orientation and basal body eccentricity in vestibular organs (utricle and saccule), three adjacent fields of 100 × 50 µm were defined centrally starting ~20 µm into the sensory region from the long edge of the organ (lateral edge in the utricle, anterior edge in the saccule). The location of these domains is illustrated in Fig. 1a (Lateral, LAT; Line of Polarity Reversal, LPR; Medial, MED in the utricle; Anterior, ANT; LPR; Posterior, POST in the saccule). These domains were selected so that the LPR domain includes hair cells of both orientations in controls. Since hair cell density and macular size are unchanged in *Gpr156* or *Emx2* mutants (Supplementary Fig. 7g,^27^), this strategy ensures that corresponding domains are compared between controls and mutants even when hair cells on one side of the LPR fail to reverse their orientation. In all circular histograms, top (‘12PM’) indicates the long edge of the organ (lateral edge in the utricle, anterior edge in the saccule). Basal body eccentricity was calculated as the ratio of the distances separating the center of the apical membrane and the PCNT-labeled basal body (bb), and the center of the apical membrane and the cell junction (radius, r) on the same axis (see Supplementary Fig. 1d). Hair cell orientation and distance values were obtained in ImageJ with the angle and straight line tools, respectively.

To measure hair cell density by region in the utricle (Supplementary Fig. 7g), three fields of 100 × 75 µm were defined centrally. The lateral extrastriolar (LES) domain was positioned ~20 µm into the sensory region from the lateral edge, and encompassed most of the region lateral to the LPR in controls. The striolar domain was defined immediately medial to the LPR in controls, and was directly adjacent to the LES domain The medial extrastriolar (MES) domain started 50 µm medial to the striolar domain. The location of these domains is illustrated in Supplementary Fig. 7g. The same domain placement and inter-domain distances were applied for *Gpr156* mutant samples lacking a LPR. The cell counter plugin in Image J was used to count hair cells by region. To measure macular surface area in the utricle (Supplementary Fig. 7g), the polygon selection and area tools in ImageJ were used using MYO7A or phalloidin to define the sensory region.

For quantification of hair cell orientation in the cochlea, either right or left cochleae were used with the field reversed (‘Flip Canvas Horizontal’ in Adobe Photoshop) as needed so that 0° (‘3PM’) on circular histograms consistently pointed towards the cochlear base. 90° (‘12PM’) indicates the lateral/abneural direction (cochlear periphery). Position analyzed along the cochlea are indicated (base, ~15%; mid, ~50%, apex ~70–75% of length starting from the base). In *Gpr156*^*del/del*^ cochlear explants, the same system was adopted to quantify the orientation of electroporated OHC1.

GPR156 protein enrichment in vestibular or cochlear organs was measured in a 30 × 10 pixel ROI window (3 × 1 or 1.5 × 0.5 µm) positioned at the hair cell junction based on ZO1 labeling. ImageJ was used to measure Integrated Density (IntDen) in the ROI in both the GPR156 and ZO1 channels at the positions indicated (Utricle: opposite the basal body vs basal body side; Cochlea: opposite the basal body vs basal body side, or medial vs lateral side). After subtracting background signal, GPR156 enrichment at each of the two opposite junctions was calculated as a ratio of the average ZO1 enrichment in the same hair cell. This value thus serves to estimate both GPR156 enrichment level and its planar asymmetry. If GPR156 IntDen was below background level, enrichment was set at 0 to avoid plotting a negative value. The utricle field where GPR156 enrichment was assessed is indicated and schematized (LPR region: 50 × 100 µm across the LPR; LES (lateral extrastriolar) region: 50 × 50 µm lateral to the LPR; Medial region: 50 × 50 µm medial to the LPR). The ImageJ Plot Profile tool was used to compare and quantify GPR156, FZD6, VANGL2, and ZO1 signals across the medial OHC junction (Fig. 8a) with a rectangle selection (averaging).

Figures were assembled using Adobe Illustrator (CS6 or CC 2020). Circular histograms reporting hair cell orientation were generated using the coord_polar function in the ggplot2 package in Rstudio (1.3.959). Other graphical charts were generated in Prism 6 or Prism 8 (GraphPad).

### Scanning electron microscopy

Temporal bones were fixed at least one overnight in 2.5% glutaraldehyde + 4% paraformaldehyde (Electron Microscopy Science) in 1 mM MgCl_2_/0.1 M Sodium Cacodylate buffer. After rinses, samples were decalcified in 0.11 M EDTA overnight, dissected in 3 pieces (cochlear base, mid, and apex), and progressively dehydrated in ethanol. Chemical drying was achieved using hexamethyldisilazane (HMDS; Electron Microscopy Science 50-243-18). Dried samples were mounted on aluminum stubs using double-sided carbon tape and sputter-coated with gold-palladium before imaging on a Hitachi 3000 N VP electronic microscope at 20 kV.

### Mouse ABR and DPOAE

Animals were tested at postnatal day (P)30 ± 2 days. Both sexes were represented and details are included in the Source Data file. DPOAE was performed two days after performing ABR on the same animals. Mice were anesthetized by intraperitoneal injection of a mix of ketamine and xylazine (10 mg and 0.1 mg per 10 g of body weight respectively) and body temperature was maintained at 37 °C using a heating pad (FHC). All tests were conducted in a sound-attenuating chamber.

For Auditory brainstem response (ABR), mice were tested using the RZ6 Multi-I/O Processor System coupled to the RA4PA 4-channel Medusa Amplifier (Tucker-Davis Technology). The TDT system was used to generate specific acoustic stimuli that included broadband clicks and 8, 16, 32 kHz pure tone bursts. One channel of ABR was recorded after binaural stimulation. Sub-dermal needles were used as electrodes. The active electrode was inserted at the vertex, the reference electrode ventrolateral to the left ear and the ground electrode to the right thigh. Auditory thresholds were obtained for each stimulus by reducing the SPL by 10 dB steps for the click and 5 dB steps for pure tones, to identify the lowest level at which an ABR could be recognized. This was done by comparing the ABR patterns with two or three suprathreshold ABRs displayed concurrently on the screen. The ABRs were typically identified with 512 stimuli presented at the rate of 21/s.

For Distortion Product Otoacoustic Emissions (DPOAEs), mice were tested using the RZ6 Multi-I/O Processor and SigGen/BioSig software (Tucker-Davis Technologies) to generate and control the stimuli. Pure tone frequencies (f2/f1 ratio = 1.2) at 8, 12, 16, and 24 kHz and at equal levels of sound pressure (L1 = L2) were generated by the RZ6 processor and attenuated through PA5 programmable attenuators. Separate drivers were used to route these attenuators to mix acoustically in the ear canal with the help of an earpiece. For each animal, sound pressures from 80 dB to 20 dB (in 10 dB decrements) were tested in 512 readings. SPLs originating from the ear canal were recorded with a low-noise prone microphone (ER 10db+ Microphone, Etymotic Research). After amplification of the signal from the microphone 10 times, the signal was re-routed to the RZ6 processor. This acoustic signal was sampled at 100 kHz and Fast Fourier Transformations (FFTs) of the signal were averaged. This FFT waveform was utilized to measure the amplitudes of f1, f2, and the (2f1-f2) distortion product (DP). Threshold for amplification was determined by comparison of the DP to background levels: if the peak of DP was higher in magnitude than any peak present in the background, acquired DP was recognized as a real signal.

### Immunoprecipitation and Western blots

HEK293 cells were cotransfected with an empty caggs vector (CMV enhancer, chicken beta-actin promoter and rabbit beta-globin splice acceptor site) or vectors expressing 2xHA, 2xHA-mouse GPR156, or untagged mouse Gαi3 using JETPrime as detailed in the manufacturer’s manual (Polyplus-transfection). After 2 days of culture, cells were collected and gently homogenized in lysis buffer for 4 h by end-to-end rotation at 4 °C (Lysis buffer: 25 mM Hepes, 2 mM EDTA, 150 mM NaCl, 10 mM NaF, 1% Triton-X100, 0.5% sodium deoxycholate, freshly made 0.1% SDS, and cOmplete protease inhibitor (SigmaAldrich 05056489001)). The lysate was then centrifuged at 4 °C for 20 min to remove debris, the input (4% volume) was set aside, and the rest of the lysate was mixed with magnetic Dynabeads Protein G (ThermoFisher Scientific 10003D) previously coated with the indicated antibody following the manufacturer’s directions. Antibodies used for IP were rabbit monoclonal anti-HA (Cell Signaling Technology 3724 S; 1:200) and chicken anti-Gαi3 (Sigma GW22489; 1:100). The cell lysis/Dynabeads mixture was incubated by end-to-end rotation overnight at 4 °C. The next day, the beads were washed 3 times for 30 minutes at 4 °C with 1 ml lysis buffer, resuspended in 60 µl lysis buffer/Laemmli (1x), and rotated overnight at 4 °C to gently detach the proteins. Standard SDS-page and immunoblotting procedures were then followed. To immunodetect proteins on blots, we used rabbit monoclonal anti-HA (Cell Signaling Technology 3724 S; 1:2000) and rabbit anti-(human) “Gαi2” (Proteintech 11136-1-AP; 1:1000). Note that anti-Gαi2 is not specific for mouse Gαi2 and also detects close homolog Gαi3, as also established in independent experiments in our laboratory.

### Zebrafish animals

Zebrafish were maintained at 30 °C using standard methods. All lines were maintained in a Tu or TL wild-type background. Larvae were raised in E3 embryo medium (5 mM NaCl, 0.17 mM KCl, 0.33 mM CaCl_2_, and 0.33 mM MgSO_4_, pH 7.2). All zebrafish work was performed at the National Institutes of Health (NIH) and was approved by the Animal Use Committee at the NIH under animal study protocol #1362-13. Larvae were examined at 5 days post fertilization (dpf). For calcium imaging in lateral-line hair bundles, the following transgenic line was used: *Tg(-6myo6b:GCaMP6s-CAAX)*^*idc1Tg*^^41^. An existing zebrafish mutant, *gpr156*^*sa34566*^ was obtained from the Zebrafish International Resource Center. This mutant results in a stop codon in the last coding exon (aa 734/797). This allele was genotyped using standard PCR and sequencing and the following primers set: FWD 5′-CCTCCGCTGGACTGATAGAG-3′ and REV-5′- GCGGTAGAAATCCTCGTCCT-3′. A CRISPR-Cas9 *gpr156* zebrafish mutant (*gpr156*^*idc15*^, denoted as *gpr156*^*exon2*^ in the figures*)* was generated using CRISPR-Cas9 technology as detailed in^60^. The second coding exon was targeted using the following guide: 5′-CAGGAGACAGAGACCGACTC (TGG)-3′. Founder fish were identified using fragment analysis of fluorescent PCR products^60^. From these founder fish, a *gpr156* mutant was identified that contained a 7 bp deletion 5′-AGCAGTGTGGAT—(GTCCAGA)—GTCGGTCTCTGTCTCCTG-3′. This 7 bp deletion results in a predicted stop codon at the middle of the third coding exon—just prior to the second transmembrane domain of Gpr156 (aa 109/797). Genotyping of this CRISPR mutant, *gpr156*^*idc15*^ was accomplished using standard PCR and sequencing using the following primers: FWD 5′-ATTTTGCCGTTTGTCTGAATCT-3′ and REV 5′-AATACAGCTCTTGCTCCTGCTC-3′.

### Zebrafish immunohistochemistry, confocal imaging and analysis

Immunohistochemistry to label actin in zebrafish hair bundles using phalloidin stain was performed on whole zebrafish larvae similar to previous work^27^. For Emx2 labeling, larvae were fixed with 4% paraformaldehyde in PBS for 3.5 hr at 4 °C. After 5 × 5 min washes in PBS + 1% DMSO, 0.5% Triton-X100, 0.1% Tween-20 (PBDTT), larvae were then blocked for 1 h at room temperature with PBDTT buffer containing 2% goat serum and 1% bovine serum albumin (BSA). Primary antibodies rabbit anti-Myosin7a (Proteus 25-6790; 1:500); mouse anti-Emx2 (Trans Genic KO609; 1:250) were diluted in PBDTT buffer containing 1% BSA and larvae were incubated in the solution overnight at 4 °C. After 5 × 5 min washes in PBDTT to remove the primary antibodies, diluted secondary antibodies (1:1000) coupled to Alexa 546 (#A21133, #A11010), or Alexa 647 (#A21241, #A21242) along with Alexa 488 Phalloidin (#A12379) (ThermoFisher Scientific) were added in PBDTT buffer containing 1% BSA and incubated for 2 hr at room temperature. After 5 × 5 min washes in PBDTT to remove the secondary antibodies, larvae were rinsed in H_2_O and mounted in Prolong gold (ThermoFisher Scientific).

Fixed zebrafish samples were imaged on an inverted Zeiss LSM 780 laser-scanning confocal microscope using confocal or Airyscan mode (Carl Zeiss AG) using an 63×/1.4 NA oil objective lens. Confocal and Airyscan z-stacks were acquired every 0.3 µm and 0.18 µm respectively. The Airyscan Z-stacks were processed with Zeiss Zen Black software v2.1 using 3D filter setting of 6.0. Experiments were imaged with the same acquisition settings to maintain consistency between comparisons. Processed imaged were further processed using Fiji. Hair bundles orientation was scored relative to the midline of the muscle somites. Hair cell number per neuromast were quantified based on Myosin7a labeling. For quantification of Emx2 labeling, hair cells were scored as Emx2 positive if they labeled with both Emx2 and Myosin7a. In each neuromast all hair cells (~14 hair cell per neuromast, Fig. 4l) were examined for our quantifications.

### Functional calcium imaging in zebrafish hair bundles

The protocol for GCaMP6s-based calcium imaging in zebrafish hair bundles is detailed in^61^. Briefly, individual 5 dpf larvae were first anesthetized with tricaine (0.03% Ethyl 3-aminobenzoate methanesulfonate salt, SigmaAldrich) and then pinned onto a Sylgard-filled recording chamber. To suppress the movement of intact larvae, alpha-bungarotoxin (125 μM, Tocris) was injected into the cavity of the heart. Larvae were immersed in extracellular imaging solution (in mM: 140 NaCl, 2 KCl, 2 CaCl_2_, 1 MgCl_2_ and 10 HEPES, pH 7.3, OSM 310±10) without tricaine. A fluid jet (HSPC-1, ALA Scientific) was used to mechanically stimulate the apical bundles of hair cells of the A-P neuromasts. To stimulate the two polarities of hair cells (A to P and P to A) a 500 ms anterior or posterior directed stimuli (anterior push or outward fluid flow; posterior pull or inward fluid flow) was applied separately.

To image calcium-dependent mechanosensation in apical hair bundles, a Bruker Swept-field confocal system was used. The Bruker Swept-field confocal system was equipped with a Rolera EM-C2 CCD camera (QImaging) and a Nikon CFI Fluor 60×/1.0 NA water immersion objective. To coordinate stimulation with image acquisition, the fluid jet was driven by a voltage-step command from the imaging software (Prairie view) during image acquisition. To simultaneously image the calcium activity in all hair bundles a piezoelectric motor (PICMA P-882.11-888.11 series, PI instruments) attached to the objective was used to allow rapid imaging in 5 planes along the Z-axis at 0.5 μm intervals, at a 50 Hz frame rate yielding a 10 Hz volume rate. The 5 plane Z-stacks were projected into one plane for image processing and quantification. The projected images were processed using a custom program with a user-friendly GUI interface in MATLAB R2014 (MathWorks). The generation of the spatial ∆F heatmaps is detailed in^61^. Briefly, we created a baseline image (F_0_ or reference image) by averaging the GCaMP6s images acquired during the pre-stimulus period. Then we subtracted this baseline image (F_0_) from each subsequent GCaMP6s image acquired, to generate an image series of the relative change (∆F) in fluorescent GCaMP6s signal from baseline. The series of ∆F images during the stimulus period was binned, scaled and encoded by a color heatmap with red indicating an increase in signal intensity. This heatmap was then overlaid onto the baseline image (F_0_). For hair bundle-localized GCaMP6s measurements, a circular ROI with a ~1.5 μm diameter was placed on the center of an individual bundle. The mean intensity (∆F/F_0_) within each ROI was then measured and plotted. The GCaMP6s signal in each hair bundle was examined to determine its functional orientation. GCaMP6s signals were examined in all hair cells in every neuromast tested. The percent of hair bundles per orientation for each neuromast were averaged for each genotype to give a P to A and A to P orientation readout.

### Statistical analysis

#### Mouse data

Throughout the study, “n” indicates the number of hair cells analyzed and “n” is indicated in the figure itself near the plot. “N” indicates the number of animals represented and is indicated in the figure legend. All values plotted in the figures are also reported along with cell sample size and animal numbers in the Source Data File (each tab corresponds to one experiment). When not quantified, all immunolabeling experiments included at least 3 mutant samples in two litters, and a similar number of littermate control animals. In that case, figure panels show a representative outcome observed in all mutant samples. Immunoprecipitation and blotting experiments were repeated 3 times to ensure that the same outcome was observed, and representative blot images are presented (Supplementary Fig. 7h-i).

All error bars indicate standard deviation. The distribution of hair cell orientation in control and mutants for the same organ/position/hair cell type was compared using Watson’s non-parametric two sample (U2) test of homogeneity. The watson.two.test function in R (circular package) was used with a significance level of a = 0.001, rejecting the null hypothesis of similar distributions when U2 > 0.385. For each comparison, the U2 value was indicated along with “*” if U2 > 0.385, or “ns” (non significant) if U2 < 0.385. Higher U2 values indicate higher significance. Because the test is sensitive and tends to lump together modestly and drastically variable distributions, we also calculated and indicated a Normalized Difference value (ND). ND represents the number of circular standard deviations (CSD) separating the circular means (CM) in the control and mutant distributions. ND was calculated in R as the angular distance between the two CMs divided by the average CSD of the two distributions. U2 and ND for each comparison along with CM and CSD for each condition are presented in the Source Data file. CM and CSD were not represented on circular histograms because they would be of limited informative value considering either uniform or bimodal (LPR macular regions) distributions and the very obvious distribution defects in mutants (reversed, and not imprecise, hair cell orientation).

Other mouse statistical analyzes were performed in Prism 6 or Prism 8 (GraphPad). Basal body eccentricity and hair cell density/macular surface area data were analyzed using an unpaired t-test (Mann-Whitney). GPR156 protein enrichment was compared using either an unpaired t-test (Mann-Whitney; to compare opposite medial and lateral enrichment per hair cell type in the wild-type cochlea), or one-way ANOVA (Kruskal-Wallis with Dunn’s multiple comparisons; to compare opposite junctional enrichments in a) control and mutant hair cells, or b) hair cells with different orientations in the wild-type utricle). ABR and DPOAEs data (pure tones) were analyzed using two-way ANOVA with Sidak’s multiple comparison test, and ABR “click” data was analyzed using unpaired Mann-Whitney test.

#### Zebrafish data

Data was plotted with Prism 8 (GraphPad). Values in the text and data with error bars on graphs and in text are expressed as mean ± SEM. All zebrafish experiments were performed on a minimum of 3 animals and 8 neuromasts. Where appropriate, datasets were confirmed for normal distributions using a D’Agostino-Pearson test. A Tukey’s multiple comparisons test was used to compare the proportion of HC orientations per neuromast between genotypes. A Mann-Whitney test or unpaired t-test was used to compare differences in HC number or proportion of Emx2^+^ HCs per neuromast as appropriate. A Sidak’s multiple comparisons test was used in our calcium imaging experiments to compare the percent of HCs of each orientation per neuromast between genotypes.

### Reporting summary

Further information on research design is available in the Nature Research Reporting Summary linked to this article.

## Supplementary information

Supplementary Information

Reporting Summary

## Data Availability

The data for all graphical representations in this article are included in the Source Data File. Additional relevant information can be obtained by contacting the authors. Source data are provided with this paper.

## Source data

Source Data
