# Supplementary material for: EMX2-GPR156-Gαi reverses hair cell orientation in mechanosensory epithelia
Source: Nat Commun. 2021 May 17;12:2861. doi: 10.1038/s41467-021-22997-1 (PMC8129141; doi:10.1038/s41467-021-22997-1)
Supplement: Supplementary file 1 — Supplementary Information [file 41467_2021_22997_MOESM1_ESM.pdf]

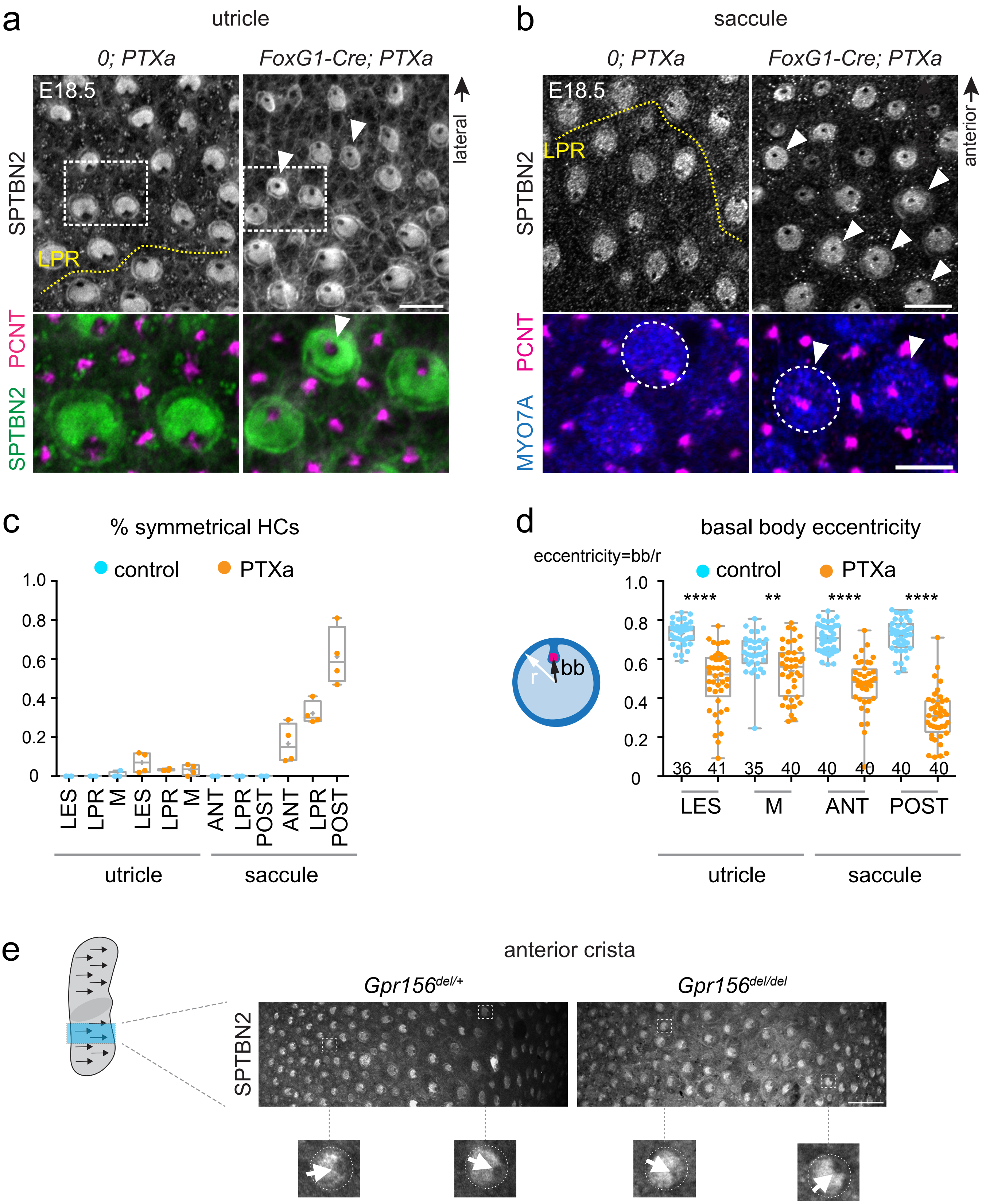

Supplementary Figure 1. Expression of Pertussis toxin catalytic subunit (PTXa) variably impairs the off-center position of the basal body in mouse macular organs. Normal hair cell orientation in *Gpr156* mutant cristae. a, b, LPR domain in the utricle (a) and saccule (b). Top panels show a low magnification view with SPTBN2 (βII-spectrin) revealing HC orientation by the position of the off-center fonticulus devoid of signal. Bottom panels show a region at higher magnification where PCNT labels the basal body below the fonticulus. The apical HC surface is labeled by SPTBN2 for boxed HCs in the top panel (a), or by MYO7A for HCs in a

different field (b). A fraction of PTXa-expressing HCs (arrowheads) have a rather central fonticulus/basal body. Because their orientation is unclear, they were omitted in the orientation analysis (Figure 1e-f). **c**, Proportion of HCs with a central basal body per domain and organ at E18.5-P0 (see Fig. 1a for a depiction of the domains). PTXa indicates the Cre-inducible *R26-LSL-PTXa* allele. Controls are Cre-negative *PTXa* animals (N=4 animals; min to max, box 25-75%). **d**, Basal body (bb) eccentricity as the ratio of the HC radius (r) for the LES and M domains (utricle) and the ANT and POST domains (saccule). The number of E18.5-P0 HCs analyzed is indicated (mean±SD; N= 4 animals; min to max, box 25-75%; Mann-Whitney test (two-tailed), **** p<0.0001; ** p=0.0024). Note how saccular HCs tend to have a more central basal body than utricle HCs, and how LES (utricle) and POST (saccule) HCs (*Emx2*$^{+}$ regions) tend to have a more central basal body compared to M (utricle) and ANT (saccule) HCs (*Emx2*$^{-}$ regions). **e**, Anterior cristae labeled with SPTBN2. HC orientation is unchanged and remains generally uniform in *Gpr156* mutants. Boxed HCs are magnified in the bottom panels, and arrows indicate HC orientation. The crista scheme indicates the domain imaged (blue). Scale bars are 10μm (a, b top), 5μm (b bottom), 20μm (e).

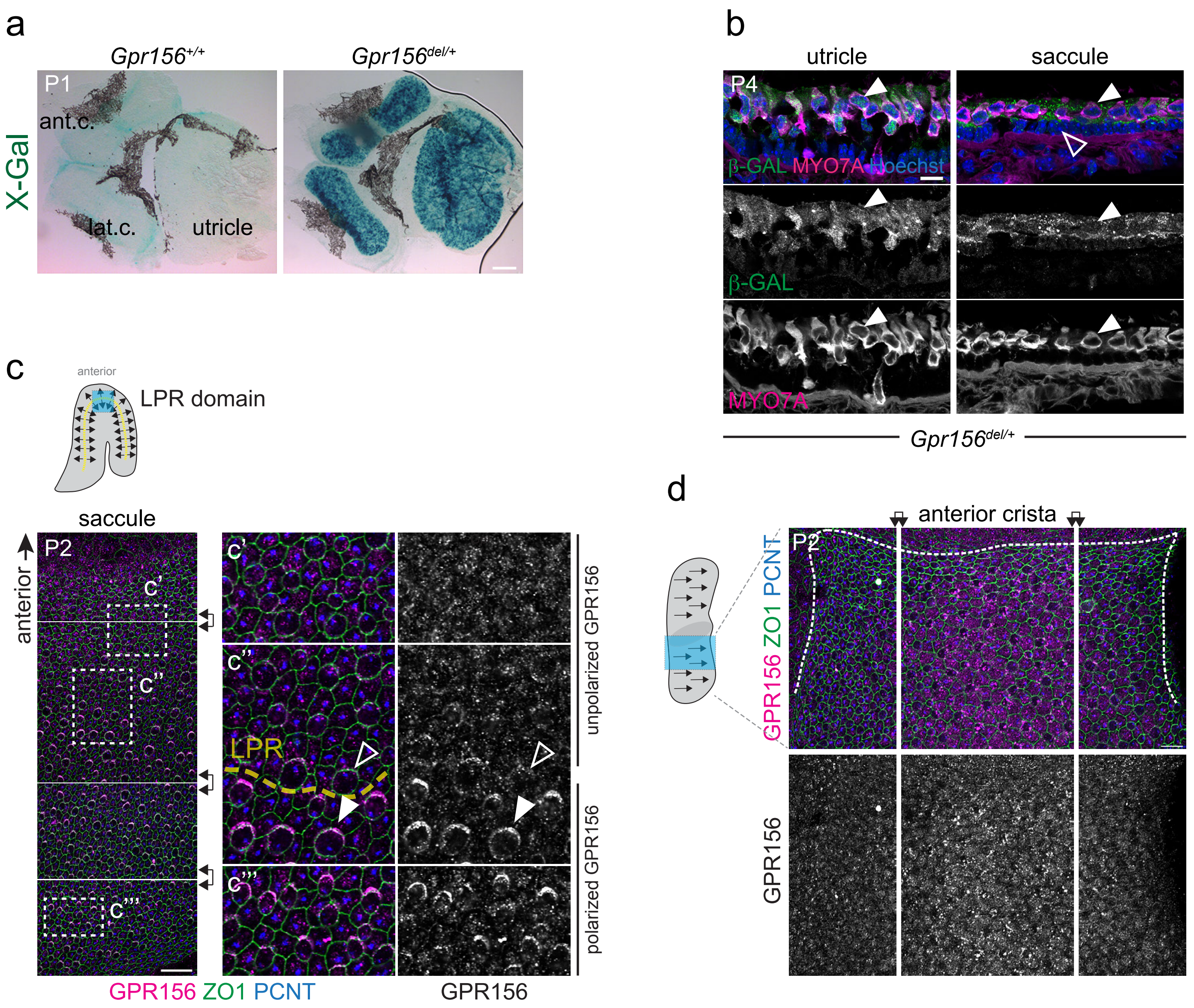

Supplementary Figure 2. *Gpr156* expression and GPR156 protein localization in mouse vestibular organs. **a,** P1 utricle, anterior (ant.) and lateral (lat.) cristae (c.) showing *LacZ* expression (X-gal signal) in *Gpr156*$^{del/+}$ (right) compared to background signal in a wild-type littermate (left). **b**, β-galactosidase (β-GAL) immunolabeling of P4 *Gpr156*$^{del/+}$ utricle and saccule cross-sections reveal *LacZ* expression. β-GAL signal is limited to MYO7A^{+} HCs (solid arrowheads). Support cells (hollow arrowhead) have no β-GAL signal. **c,** P2 wild-type saccule where basal body labeling (PCNT) indicates HC orientation. GPR156 polarization (solid arrowheads) is limited to posterior HCs oriented posteriorly. HCs across the LPR oriented anteriorly do not show polarized GPR156 (hollow arrowheads). Boxed regions in continuous fields in the left panels are magnified in the central and right panels. **d,** P2 wild-type anterior crista. GPR156 is not planar polarized. Left to right panels show continuous fields across the crista on one side of the eminentia cruciata (illustrated on the left cartoon). Note that cristae are *Emx2*$^{-}$ and do not have a LPR. In c and d, the schemes indicate the domain imaged (blue). Scale bars are 100µm (a), 10µm (b, d), 20µm (c).

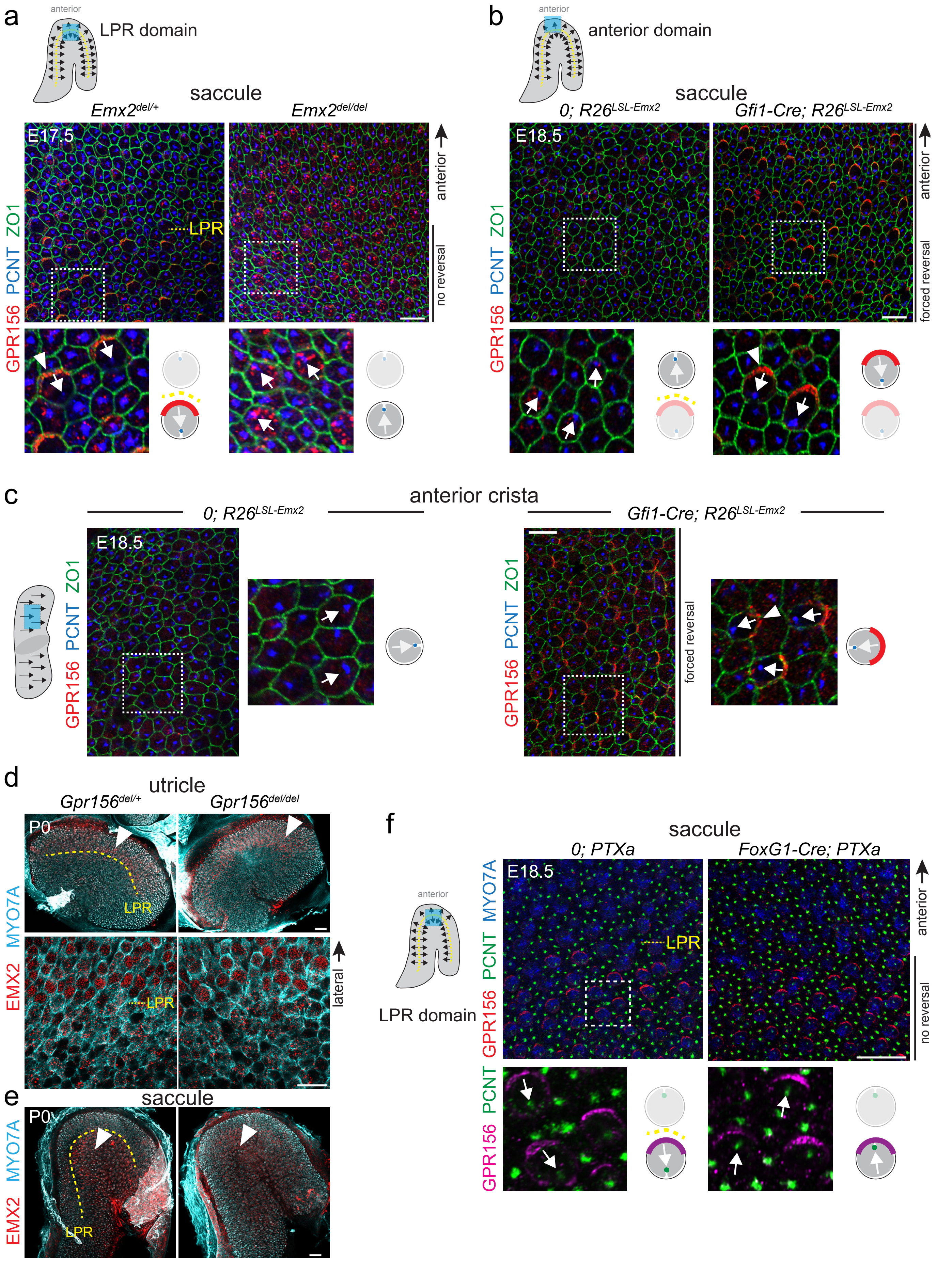

Supplementary Figure 3. EMX2 > GPR156 > Gαi epistasis in mouse macular organs. a, LPR domain in

E17.5 saccules. Polarization of GPR156 in posterior HCs (arrowhead) is lost when EMX2 is missing and these HCs fail to reverse. **b,** Anterior domain in E18.5 saccules. Ectopic expression of *Emx2* reverses HC orientation and induces polarization of GPR156 (arrowhead) in anterior HCs. **c**, E18.5 Anterior crista. Ectopic expression of *Emx2* reverses HC orientation and induces polarization of GPR156 (arrowhead) in crista HCs. **d-e,** P0 utricles (d) and saccules (e) labeled with EMX2 and MYO7A. EMX2 zonal expression in the lateral utricle (d, arrowheads) and posterior saccule (e, arrowheads) is unchanged when GPR156 is missing and HCs in these compartments fail to reverse (see Figure 1). Bottom panels in d show higher magnification views where nuclear EMX2 labeling ends at a position corresponding to the LPR in controls. **f,** LPR region in E18.5 saccules. Polarization of GPR156 in posterior HCs expressing PTXa is intact although these HCs fail to reverse. In a-c, f, vestibular organs are labeled with GPR156, PCNT and ZO1 (a-c) or MYO7A (f). Boxed areas are magnified in the lower panels, and HC orientation and GPR156 distribution is summarized in a cartoon form. Arrows indicate HC orientation based on PCNT-labeled basal body. Saccule or crista schemes indicate the domain imaged (blue). Yellow dashed lines represent the Line of Polarity Reversal (LPR) in controls. Scale bars are 10µm (a-c), 50µm (d top, e), 20µm (d bottom, f).

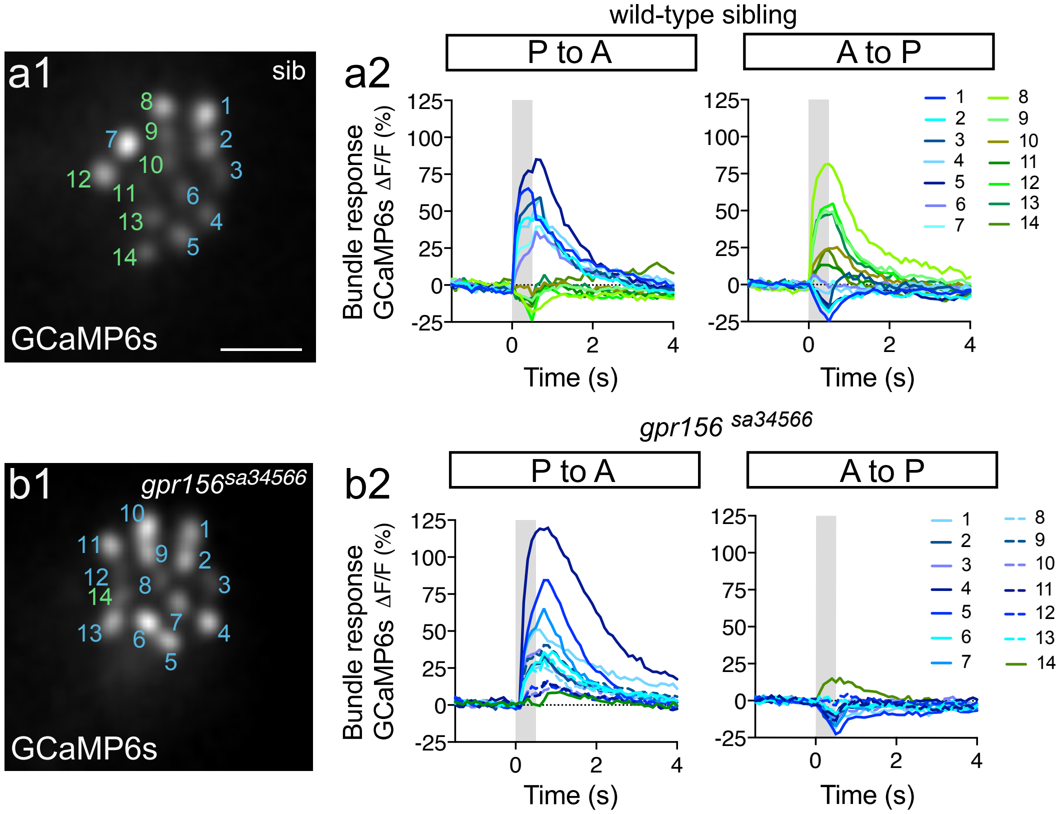

Supplementary Figure 4. Examples of temporal GCaMP6s mechanosensitive calcium responses in individual wild-type and *gpr156* mutant hair bundles. a1, b1, The hair bundles expressing GCaMP6s shown in a1 and b1 are the same as the ones depicted in Figure 4o1, 4p1. Numbered HCs in a1 and b1 correspond to the individual response traces in a2 and b2, respectively. **a2**, **b2**, While a similar number of hair bundles respond to P to A (blue traces) and A to P (green traces)-directed stimuli in wild-types, in *gpr156* mutants, the majority of HCs respond to P to A directed stimuli. Scale bar is 5μm (a1 and b1).

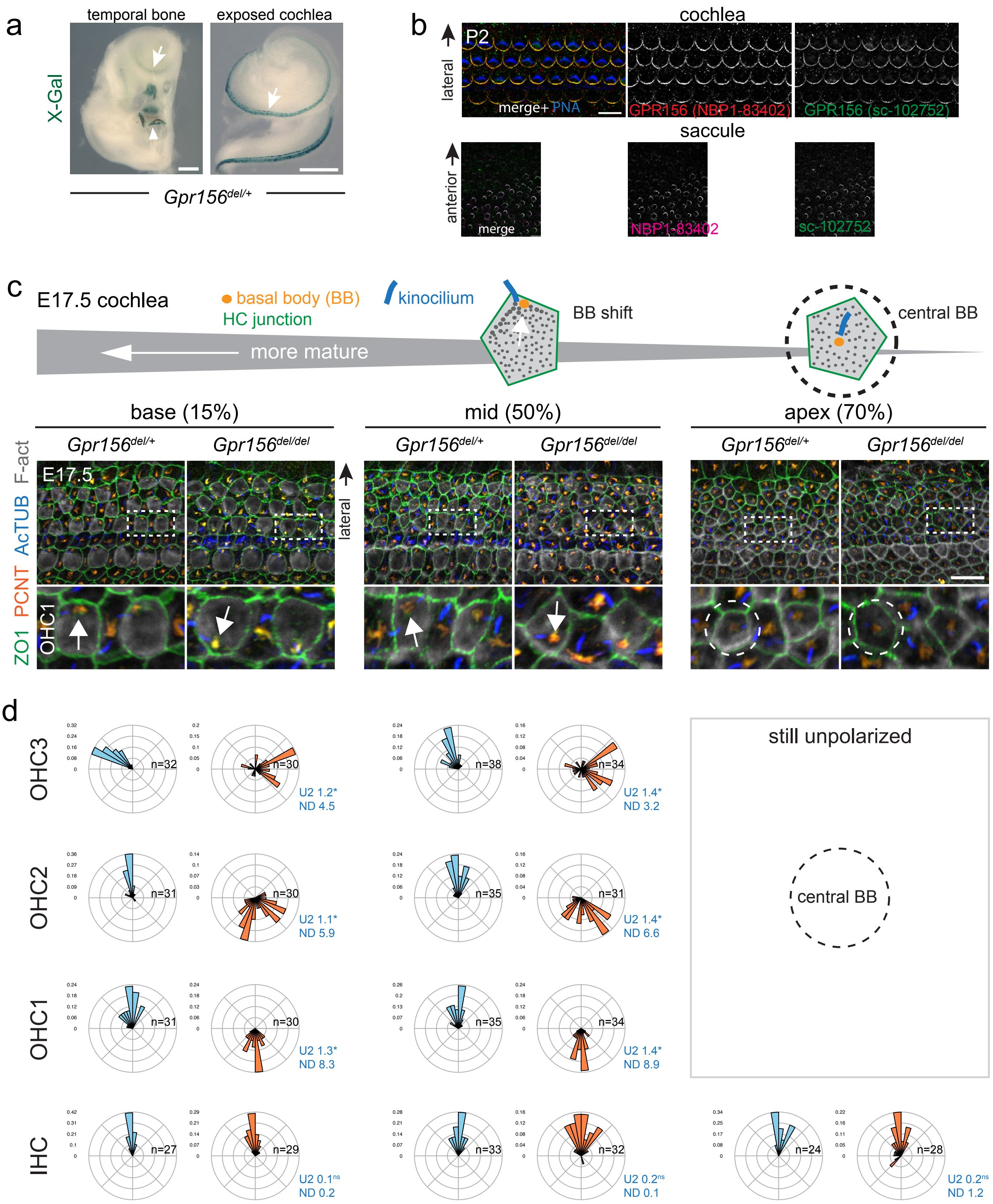

Supplementary Figure 5. GPR156 expression and E17.5 *Gpr156* mutant phenotype in the mouse auditory epithelium. a, Stereomicroscope views of *Gpr156*$^{del/+}$ whole inner ear (left) and dissected cochlea (right) where *LacZ* reporter expression is limited to sensory regions. Arrows indicate the auditory epithelium, arrowhead indicates vestibular organs. **b,** Co-labeling with goat anti-GPR156 (sc-102752, Santa Cruz Biotechnology) and rabbit anti-GPR156 (NBP1-83402, Novus). Similar protein polarization patterns are observed in the cochlea (top) and saccule (bottom) for both antibodies. Peanut agglutinin (PNA) labels hair bundles in OHCs. **c,d**, Evidence

for timely but inverted basal body (BB) shift in *Gpr156* mutants compared to controls. 3 positions along the maturing E17.5 cochlea represent pseudo-time (apex, 70%, prior to basal body shift in OHCs; mid, 50%, shortly after basal body shift in OHCs; base, 15%, most mature). **c**, E17.5 auditory epithelium. The basal body (PCNT) and kinocilium (AcTUB) are labeled relative to cell outlines (ZO1) and F-actin. Note how shortly after the basal body shift (at 50%), mutant OHC1s (magnified insets) already have a medial basal body/kinocilium, and thus a medial orientation contrasting with a lateral basal body and lateral orientation in controls (arrows). Slightly more apical OHCs (at 70%) have a central basal body/kinocilium and are thus still unpolarized (dashed circle) in both genotypes. **d**, Circular histograms of E17.5 HC orientation by row. Histograms show frequency distribution at the cochlear positions indicated in c (10° bins in a referential where 90° (top) is lateral and 0° (right) is towards the cochlear base; n indicates HC number in 4 animals; Watson U2 test of homogeneity; normalized difference (ND) value indicates how many standard deviations separate the circular means of each distribution). Arrows indicate HC orientation. Scale bars are 0.5mm (a), 10µm (b-c).

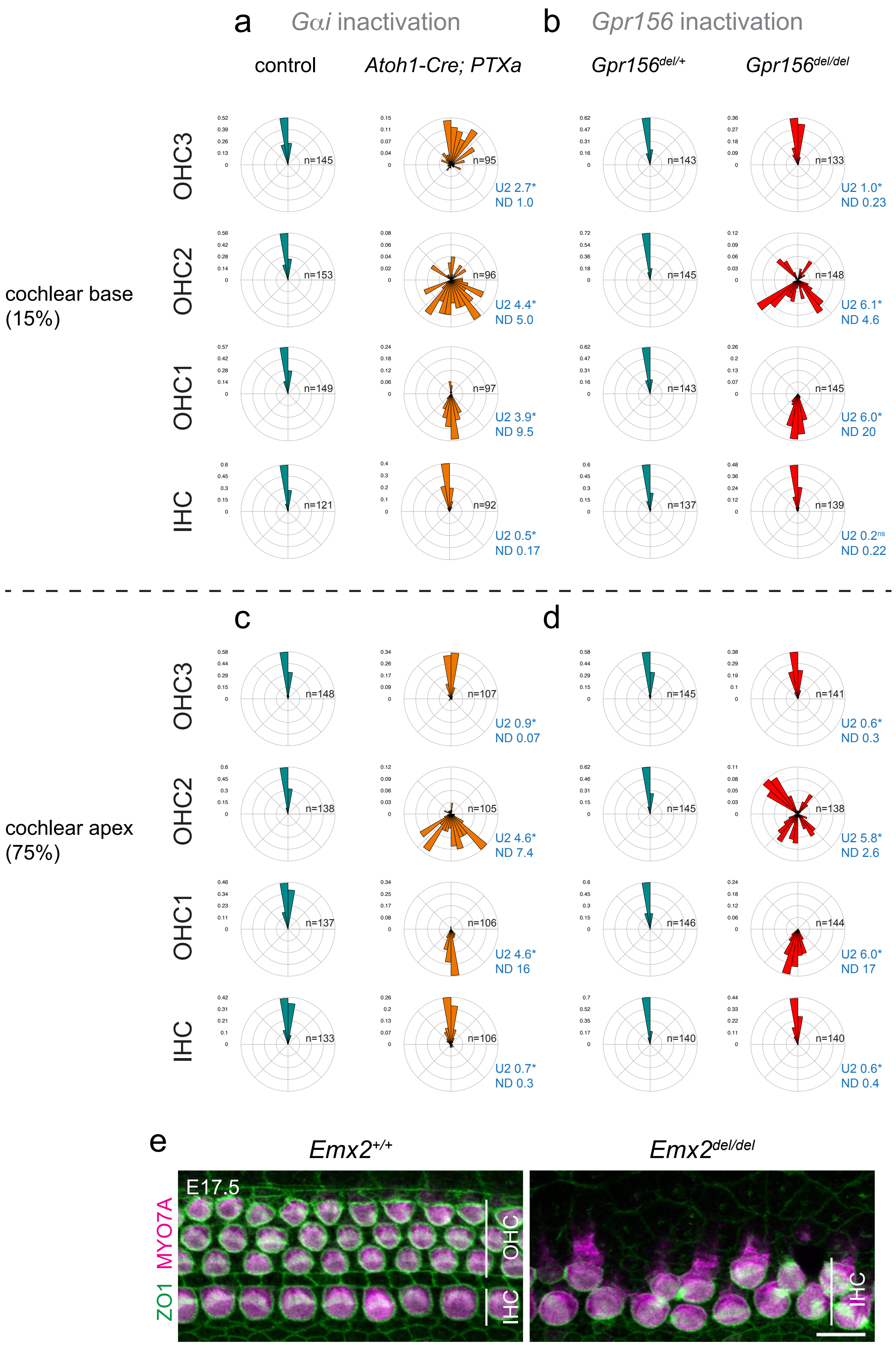

Supplementary Figure 6. Cochlear hair cell orientation defects upon Gαi or *Gpr156* inactivation at base and apex positions. Loss of outer hair cells in a new *Emx2* mutant. a-d, Circular histograms of P4 HC orientation by row. Histograms show frequency distribution at 15% (**a-b**, base, most mature) and 75% (**c-d**, apex,

less mature) cochlear positions (10° bins in a referential where 90° (top) is lateral and 0° (right) is towards the cochlear base; n indicates HC number in 5-7 animals; Watson U2 test of homogeneity; normalized difference (ND) value indicates how many standard deviations separate the circular means of each distribution). A pattern of graded OHC inversion by row is shared in *PTXa* (*Atoh1-Cre* driver; a, c) and *Gpr156* (b, d) mutants. *PTXa* indicates the Cre-inducible *R26-LSL-PTXa* allele. Littermate controls for *Atoh1-Cre*; *PTXa* are Cre-negative *PTXa* animals. **e**, E17.5 auditory epithelium. MYO7A and ZO1 co-labeling shows loss of OHCs in *Emx2* mutants. Scale bar is 10μm (e).

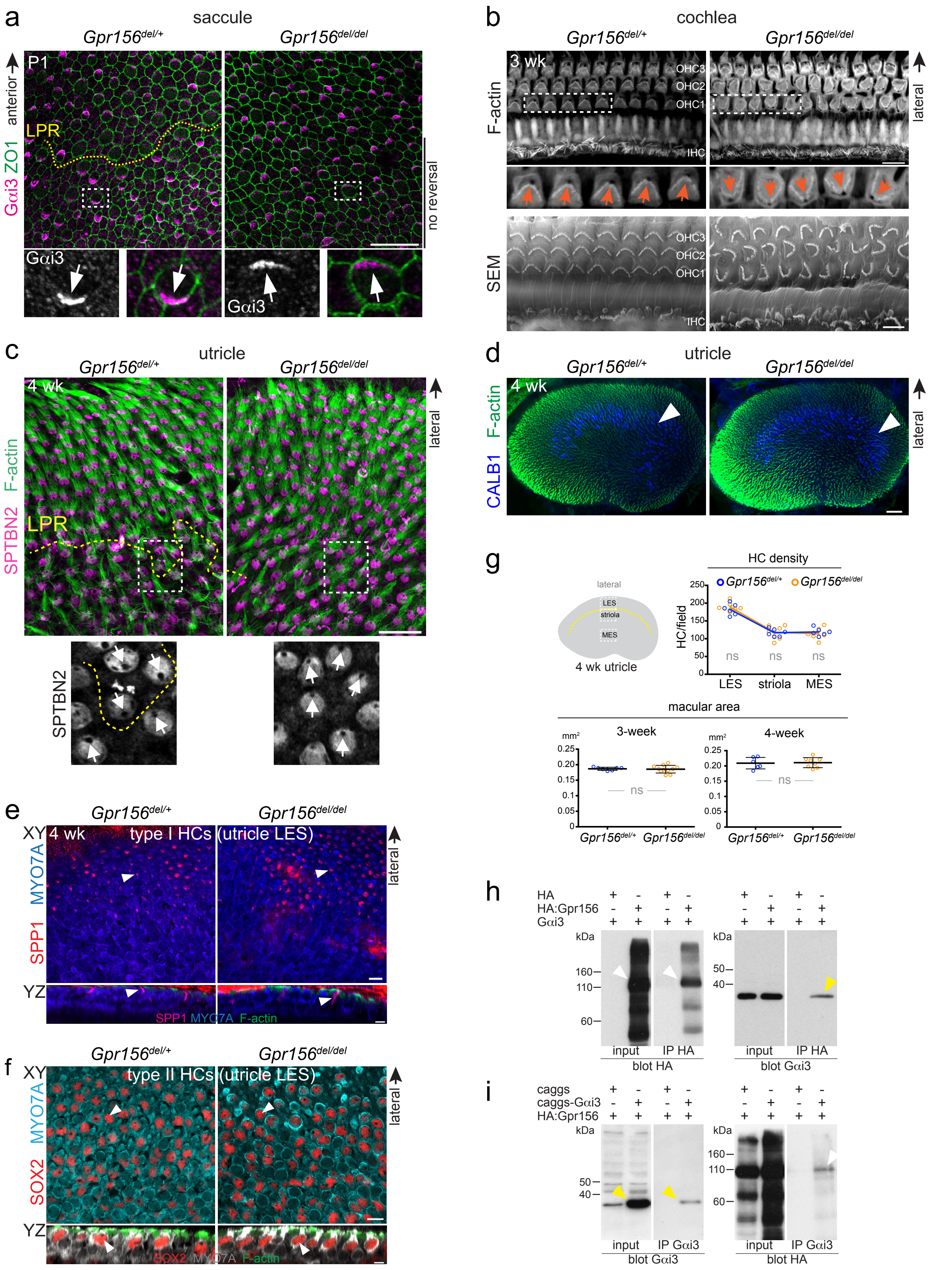

Supplementary Figure 7. GPR156-Gαi and GPSM2-Gαi are distinct functional modules in developing

hair cells. Mature *Gpr156* mutants have no obvious inner ear defects besides hair cell misorientation. GPR156 and Gαi3 protein interact. a, Normal apical enrichment of Gαi3 in P1 *Gpr156* mutant HCs in the saccule. Arrows indicate HC orientation. Note how Gαi3 enrichment follows HC orientation when HCs fail to reverse orientation (posterior HCs). Bottom panels show a magnified view of the HC boxed in the upper panels. **b,** Phalloidin labeling (F-actin, top) and scanning electron microscopy (SEM, bottom) of the auditory epithelium at 3 weeks at the middle cochlear turn. Adult *Gpr156* mutants retain graded OHC inversion observed at E17.5 and P0-P4 (Supplementary figure 5c; Figure 5d), and do not show HC death. Bottom panels show a magnified view of the OHC1s boxed in the upper panels. **c,** LPR region in the utricle at 4 week. SPTBN2 (βII-spectrin) reveals HC orientation (arrows) by the position of the off-center fonticulus devoid of signal. The LPR (dashed yellow line) can be traced in controls but not in mutants, where lateral HCs failed to reverse and generally point laterally. Bottom panels show the SPTBN2 channel at higher magnification for the boxed region. **d,** Utricle at 4 week. Calbindin1 (CALB1) labeling of striolar afferents reveals a normal striolar region (arrowheads) in *Gpr156* mutants. **e, f,** Normal distribution of type I (e, $SPP1^+$) and type II (f, $SOX2^+$) HCs in the lateral extrastriolar (LES) region of *Gpr156* mutant utricle. Top and bottom panels show XY and YZ views, respectively. SPP1 labels the type I HC neck region (e, arrowheads) and SOX2 labels type II HC nuclei (f, arrowheads). **g,** Utricle at 3-4 week. HC density (top) in the domain outlined in the left scheme and total sensory surface area (bottom). *Gpr156* mutants have no obvious defects in HC number or density (mean±SD; HC density: N= 6 $Gpr156^{del/+}$ animals per domain, N=8 $Gpr156^{del/del}$ per domain; Surface area: N=7 $Gpr156^{del/+}$ animals at 3 week, N=6 at 4 week, N=11 $Gpr156^{del/del}$ at 3-week, N=8 at 4 week. Both sex are represented. Mann-Whitney test (two-tailed), ns is p=0.4336 (LES HC density), p=0.6863 (STR HC density), p=0.5941 (MES HC density), p=0.8424 (area at 3-week), p=0.9497 (area at 4-week)). LES and MES, lateral and medial extrastriolar domains, respectively. **h**, Co-immunoprecipitation of Gαi3 (yellow arrowhead) with HA-GPR156 (white arrowheads), but not with HA, in HEK293 cells. **i**, Co-immunoprecipitation of HA-GPR156 (white arrowhead) with Gαi3 (yellow arrowheads), but not upon empty vector (caggs) transfection. Arrows indicate HC orientation. Wk, week (age), kDa, kilodalton. Scale bars are 20µm (a, c), 10µm (b, e top, f top), 100µm (d), 5µm (e bottom, f bottom).

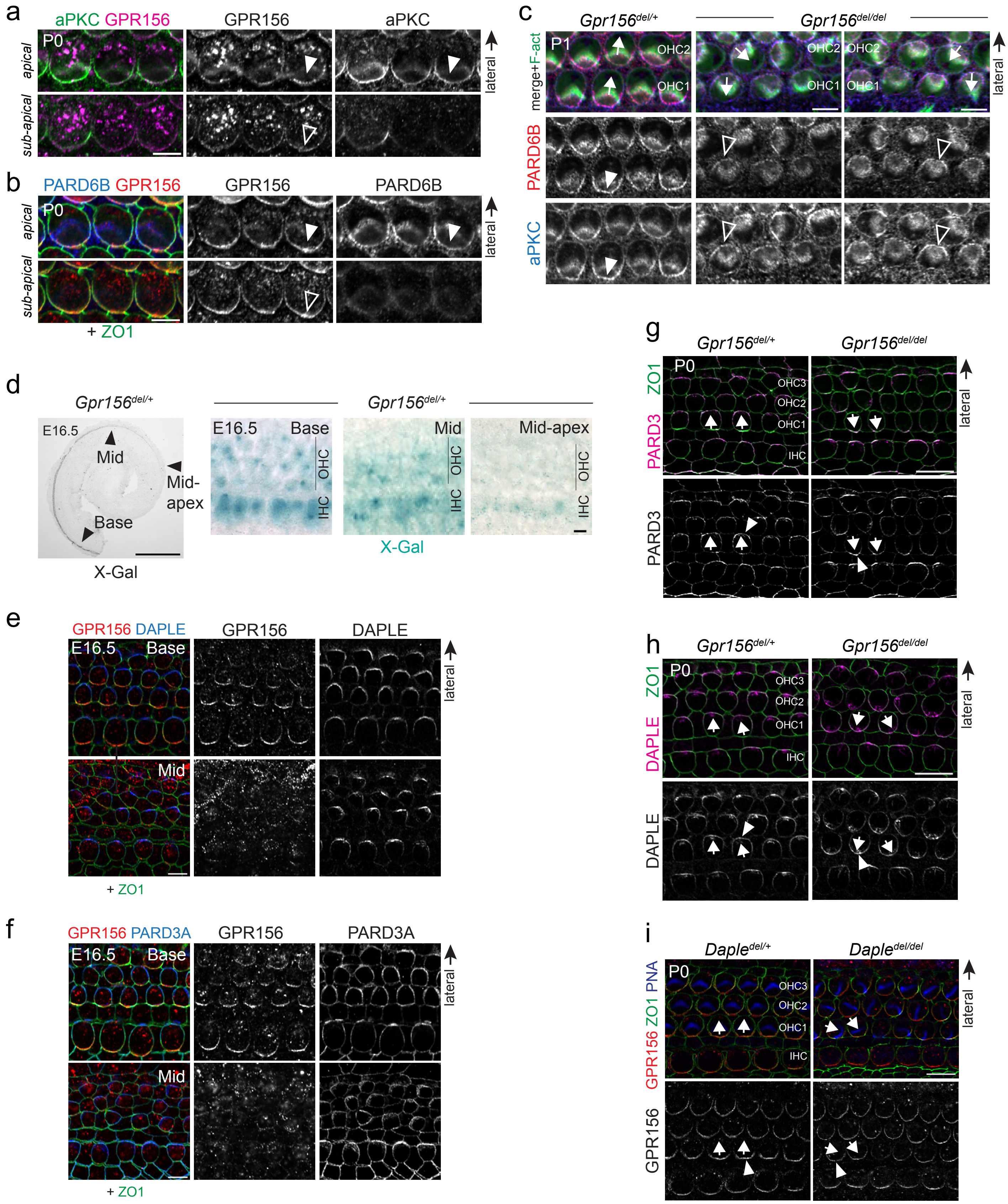

Supplementary Figure 8. Relationship between GPR156 and other polarity factors in the cochlea. a-b, GPR156 and aPKC (a) or PARD6B (b) co-labeling in wild-type P0 OHC2s. GPR156 and aPKC (a) or PARD6B (b) colocalize apically at the medial HC junction (solid arrowheads). A sub-apical view of the same field (0.5µm more basally; bottom panels) shows that GPR156 and ZO1 (hollow arrowheads) extend more basally than

aPKC and PARD6B. **c,** aPKC and PARD6B co-labeling in P1 *Gpr156*^{del} cochleae. Asymmetric aPKC-PARD6B enrichment in OHCs is retained but more variable in *Gpr156* mutants. When visible, protein polarization follows OHC misorientation so that it remains on the OHC side opposite from the vertex of the chevron-shaped hair bundle. This results in enrichment at the lateral OHC junction (hollow arrowheads) instead of the medial OHC junction in controls (solid arrowheads) when HCs are inverted (arrows). **d**, *LacZ* reporter expression in E16.5 *Gpr156*$^{del/+}$ cochlea at the positions shown on the left panel (Base, Mid, Mid-apex). *Gpr156* expression is graded in intensity along the cochlea, and only detected in IHCs at the Mid-apex position. **e-f**, GPR156 co-labeling with DAPLE (e) and PARD3 (f) in E16.5 wild-type cochleae. DAPLE and PARD3 appear to be polarized at the lateral HC junction before GPR156 can be detected at the medial HC junction (see Mid position panels; *Gpr156* is expressed there based on X-gal signals in d). **g**, PARD3 labeling in P0 *Gpr156*^{del} cochlea. **h**, DAPLE labeling in *Gpr156*^{del} cochlea. Asymmetric PARD3 and DAPLE junctional enrichment (arrowheads) is preserved in *Gpr156* mutants and follows HC misorientation, remaining in register with the HC cytoskeleton. **i**, GPR156 labeling in P0 *Daple*^{del} cochlea. GPR156 enrichment at the medial HC junction (arrowheads) is intact in *Daple* mutants in spite of HC misorientation. Arrows indicate HC orientation. PNA, peanut agglutinin to label the hair bundle in OHCs. Scale bars are 5μm (a-f), 500μm (d, left), 10μm (g-i).

Primers used for mouse genotyping by strain

Gpr156	Gpr156 F	AACCTGCGTGTGCATGTTTG	wild-type	575bp
			del	432 bp
	Gpr156 R	TCTACCACTACCACCATCAC		
	BetaGeo Rb	TCTCCCAATCTCTCCTCTGC		

R26-LSL-PTXa	R26_1b	GCACTTGCTCTCCCAAAGTC	wild-type	617bp
			knock-in	707bp
	R26-3	GGA GCG GGA GAA ATG GAT ATG		
	PGK R	GAGACGTGCTACTTCCATTTGTC		

Emx2	Emx2_ex1utrFb	TCTTCCTTCCTTCCTTTCCTTACC	wild-type	747bp
			del	~220bp
	Emx2_in1Rb	ACCCAAACGAGCTTCAGAGAC		

R26-LSL-Emx2	R10	CTCTGCTGCCTCCTGGCTTCT	wild-type	311bp
	R11	CGAGGCGGATACAAGCAATA		
	R425	GGTGATAGG TGGCAAGTGGTATTC	knock-in	369bp
	pCA-R2	GGCTAT GAACTAATGACCCCGT		

Daple	Ccdc88c_F	GTATGTGAGTGGTAAAGGCC	wild-type	795bp
	Ccdc88c_R	CAATCGTTTCTGCTTGGTGG		
	Ccdc88c_3'R	GCGTCTGACATCTGGAGACACG	del	539bp
	LacZ_F	GCTACCATTACCAGTTGGTCTGGTGTC		

FoxG1-Cre	FoxG1 Fb	GCGACAAGAAGAACGGCAAG	wild-type	202bp
	FoxG1 R	AAGCACTTGTTGAGGGACAG		
	Cre F	GCAAGAACCTGATGGACATG	Cre	381bp
	Cre R	TTTCCATGAGTGAACGAACC		

Gfi1-Cre	FWD	CACCCTGTTACGTATAGCCG	Cre	~330bp
	REV	GAGTCATCCTTAGCGCCGTA		

Atoh1-Cre	Math1 enh F	AGAGCGGCTGACAATAGAGG	Cre	~850bp
	Cre R	TTTCCATGAGTGAACGAACC		

Primers used for zebrafish genotyping by strain

gpr156 sa34566	FWD	CCTCCGCTGGACTGATAGAG	wild-type	191bp
	REV	GCGGTAGAAATCCTCGTCCT		

gpr156 idc15	FWD	ATTTTGCCGTTTGTCTGAATCT	wild-type	398bp
(*gpr156 exon 2*)				
	REV	AATACAGCTCTTGCTCCTGCTC		

Supplementary Table 1. Primers used for genotyping.
