# Supplementary material for: EMX2-GPR156-Gαi reverses hair cell orientation in mechanosensory epithelia
Source: Nat Commun. 2021 May 17;12:2861. doi: 10.1038/s41467-021-22997-1 (PMC8129141; doi:10.1038/s41467-021-22997-1)
Supplement: Supplementary file 2 — Reporting Summary [file 41467_2021_22997_MOESM2_ESM.pdf]

nature research

Corresponding author(s): basile tarchini

Last updated by author(s): Mar 17, 2021

Reporting Summary

Nature Research wishes to improve the reproducibility of the work that we publish. This form provides structure for consistency and transparency in reporting. For further information on Nature Research policies, see our Editorial Policies and the Editorial Policy Checklist.

Statistics

For all statistical analyses, confirm that the following items are present in the figure legend, table legend, main text, or Methods section.

n/a	Confirmed	
☐	☒	The exact sample size (*n*) for each experimental group/condition, given as a discrete number and unit of measurement
☐	☒	A statement on whether measurements were taken from distinct samples or whether the same sample was measured repeatedly
☐	☒	The statistical test(s) used AND whether they are one- or two-sided<br>*Only common tests should be described solely by name; describe more complex techniques in the Methods section.*
☐	☒	A description of all covariates tested
☐	☒	A description of any assumptions or corrections, such as tests of normality and adjustment for multiple comparisons
☐	☒	A full description of the statistical parameters including central tendency (e.g. means) or other basic estimates (e.g. regression coefficient) AND variation (e.g. standard deviation) or associated estimates of uncertainty (e.g. confidence intervals)
☐	☒	For null hypothesis testing, the test statistic (e.g. *F*, *t*, *r*) with confidence intervals, effect sizes, degrees of freedom and *P* value noted<br>*Give P values as exact values whenever suitable.*
☒	☐	For Bayesian analysis, information on the choice of priors and Markov chain Monte Carlo settings
☒	☐	For hierarchical and complex designs, identification of the appropriate level for tests and full reporting of outcomes
☒	☐	Estimates of effect sizes (e.g. Cohen's *d*, Pearson's *r*), indicating how they were calculated

Our web collection on statistics for biologists contains articles on many of the points above.

Software and code

Policy information about availability of computer code

Data collection	No software was directly used to collect anatomical and molecular results. Carl Zeiss Zen Blue 2.3 and 2.6 softwares were used to capture confocal microscopy images in mouse. Carl Zeiss Zen 2.3 SP1 black was use to acquired Airyscan images in zebrafish, Carl Zeiss Zen 2.3 Blue was used to process Airyscan images. Image J/Fiji (2.0.0; NIH) was used to analyze images. To collect ABR/DPOAE signals, we used the Tucker-Davis Technology system and software (SigGen/BioSig 5.7.3 (2018)).
Data analysis	Prism 6 and Prism 8 (GraphPad). RStudio 1.3.959 with package "circular"(0.4-93) for Circular statistics and package "ggplot2"(3.3.2) for circular histograms. Adobe Photoshop and Adobe Illustrator (CS6 and creative cloud 2020). Matlab 2014b was used to process the calcium imaging data.

For manuscripts utilizing custom algorithms or software that are central to the research but not yet described in published literature, software must be made available to editors and reviewers. We strongly encourage code deposition in a community repository (e.g. GitHub). See the Nature Research guidelines for submitting code & software for further information.

Data

Policy information about availability of data

All manuscripts must include a data availability statement. This statement should provide the following information, where applicable:

- Accession codes, unique identifiers, or web links for publicly available datasets
- A list of figures that have associated raw data
- A description of any restrictions on data availability

The data for all graphical representations in this article are included in the Source Data File. Additional relevant information can be obtained by contacting the corresponding author.

Field-specific reporting

Please select the one below that is the best fit for your research. If you are not sure, read the appropriate sections before making your selection.

[x] Life sciences [ ] Behavioural & social sciences [ ] Ecological, evolutionary & environmental sciences

For a reference copy of the document with all sections, see nature.com/documents/nr-reporting-summary-flat.pdf

Life sciences study design

All studies must disclose on these points even when the disclosure is negative.

Sample size	Sample size was based on comparable studies in the field and our own previous published studies (Jiang et al., Elife 2017; PMID 33377867; Lukasz and Kindt J Vis Exp 2018; PMID 30582593; Tarchini et al., Development 2016; PMID 27660326; Tadenev et al., Curr Biol; PMID 30827920). These numbers give sufficient statistical power to avoid both Type 1 and Type 2 error. For anatomical and molecular results, we observed low or no variability in the phenotypes among animals of the same genotype, and a sample size of 24<n<~200 cells per genotype/organ region/genotype representing 3<N<8 animals was used depending on the context and the experiment. For ABR/DPOAE tests where physiological measurement are made per animal, we also observed low variability among animals of the same genotype (replicates) and we used 7<N<26 animals per genotype per test type. For zebrafish studies we used a sample size of ~112<n<250 cells per genotype representing 3<N<16 animals per analysis.
Data exclusions	No data exclusion
Replication	Multiple mice were used in quantified experiments to ensure that phenotypes could be replicated. For anatomical and molecular results, we observed low or no variability in the phenotypes among animals of the same genotype, and a sample size of 24<n<~200 cells per genotype/organ region/genotype representing 3<N<8 animals was used depending on the context and the experiment. For ABR/DPOAE tests where physiological measurement are made per animal, we also observed low variability among animals of the same genotype (replicates) and we used 7<N<26 animals per genotype per test type. When not quantified, all immunolabeling experiments included at least 3 mutant samples in two litters, and a similar number of littermate control animals. In that case, figure panels show a representative outcome observed in all mutant samples. Zebrafish immunohistochemistry experiments were replicated with two independent immunostains for each gpr156 allele (Emx2 staining, hair cell counts and hair cell orientation). The zebrafish functional calcium imaging result was verified on two independent days. As all measurements correspond to tissues extracted from or examined on an animal, there are no technical replicates, only biological replicates. All findings submitted in the manuscript were replicated successfully in all animals analyzed. Immunoprecipitation/immunoblotting experiments were repeated 3 times with similar outcomes, and one representative blot is shown for each experiment.
Randomization	All experimental groups consisted of different genotypes among littermate animals. Allocation was based on PCR-based genotyping. In some cases, but not others, analyzes were done blind to the genotype because samples were collected and analyzed before their genotype was determined (see below).
Blinding	When possible analyzes were done blind to the genotype (ABR, microscopy analysis of samples for anatomical or immunostaining results). In other cases this was not realistic or possible. For example, mutants in some strains were grossly dysmorphic or had hair cell reversal defects, which revealed the genotype of the sample. For ABR and DPOAE, two investigators performed the experiments and compared results to ensure consistent conclusions. This approach was not realistic for anatomical and molecular analyzes than spanned several years and hundreds of individual samples.

Reporting for specific materials, systems and methods

We require information from authors about some types of materials, experimental systems and methods used in many studies. Here, indicate whether each material, system or method listed is relevant to your study. If you are not sure if a list item applies to your research, read the appropriate section before selecting a response.

Materials & experimental systems

n/a	Involved in the study
[ ]	[x] Antibodies
[ ]	[x] Eukaryotic cell lines
[x]	[ ] Palaeontology and archaeology
[ ]	[x] Animals and other organisms
[x]	[ ] Human research participants
[x]	[ ] Clinical data
[x]	[ ] Dual use research of concern

Methods

n/a	Involved in the study
[x]	[ ] ChIP-seq
[x]	[ ] Flow cytometry
[x]	[ ] MRI-based neuroimaging

Antibodies

Antibodies used	Primary antibodies used for tissue immunolabeling were: goat anti-GPR156 (Santa Cruz; sc102752; TCA; 1:100), rabbit anti-GPR156 (Novus; NBP1-83402; TCA; 1:100), mouse anti-acetylated Tubulin (Santa Cruz; 23950; PFA; 1:500), rabbit anti-pericentrin/PCNT

(Biolegend; PRB-432C; PFA; 1:400), mouse anti-betaII-Spectrin/SPTBN2 (BD Transduction Lab; 612562, PFA; 1:200), rat anti-ZO1 (Developmental Studies Hybridoma Bank; R26.4C; TCA; 1:200), rabbit anti-Gi3 (Santa Cruz; sc-262; PFA; 1:400), chicken anti-Gi3 (Sigma; GW22489; PFA; 1:400; used for cochlear explants), rabbit anti-DAPLE/CCDC88C (Proteintech; 25769-1-AP; TCA; 1:400), mouse anti-MYO7A (Developmental Studies Hybridoma Bank; 138-1; PFA; 1:500), goat anti-FZD6 (R&D Systems; AF1526; PFA; 1:200), rabbit anti-VANGL2 (gift from Philippe Gros, McGill University; PFA; 1:500), goat anti-GPSM2/LGN (ThermoFisher Scientific; PA5-18646; PFA; 1:200), rabbit anti-betaGalactosidase (Cappel discontinued aliquot; PFA; 1:1000; now MP Biomedical 55976), mouse anti-aPKC/PRKCZ (Santa Cruz; sc-216; PFA; 1:100), rabbit anti-PARD6B (Santa Cruz; sc-67393; PFA; 1:100), rabbit anti-PARD3A (Proteintech; 11085-1-AP; PFA; 1:200), rabbit anti-CALB1 (Cedarlane/Millipore; AB1778(CH); PFA; 1:500), goat anti-SPP1/Osteopontin (R&D Systems; AF808; PFA; 1:100), goat anti-SOX2 (Santa Cruz; 17320; PFA; 1:500), rabbit anti-EMX2 (Trans Genic; KO609; PFA; 1:250), rabbit anti-Myosin7a (Proteus 25-6790; 1:500). Secondary antibodies were raised in donkey or goat and coupled to Alex Fluor (AF) 488 (used 1:1000), 555 (used 1:1000) or 647 (used 1:500) (ThermoFisher Scientific). Fluorescent conjugated phalloidins were used to reveal F-actin (ThermoFisher Scientific: AF488, A12379; AF555, A34055. Biotium: CF405, 89138-126).

Antibodies used for immunoprecipitation were rabbit monoclonal anti-HA (Cell Signaling Technology 3724S; 1:200) and chicken anti-Gi3 (Sigma GW22489; 1:100). Antibodies used for immunoblotting were rabbit monoclonal anti-HA (Cell Signaling Technology 3724S; 1:2000) and rabbit anti-(human) Gi2 (Proteintech 11136-1-AP; 1:1000).

Validation

The GPR156 antibodies were validated in this study using immunolabeling in tissue samples from Gpr156 mutants lacking the protein. Signal observed in control littermate but lost in mutants was deemed specific. Other important antibodies and protein localization patterns have been validated previously. For example, rabbit anti-Gi3 and goat anti-GPSM2/LGN (Tadenev et al., Curr Biol; PMID 30827920), rabbit anti-DAPLE (Siletti et al., PNAS 2017; PMID 29229865), rabbit anti-EMX2, mouse anti-betaII-Spectrin (Jiang et al., Elife 2017; PMID 33377867), goat anti-FZD6, mouse anti-aPKC (Tarchini et al., Dev Cell 2013; PMID 24135232), rabbit anti-PARD6B (Ezan et al., Nat Cell Biol 2013; PMID 23934215), rat anti-ZO1 (Marivin et al., JCB 2019; PMID 30948426), rabbit anti-PCNT (Stoller et al., Dev Biol 2018; PMID 29510119).

Eukaryotic cell lines

Policy information about cell lines

Cell line source(s)

ATCC (HEK293 CRL-1573)

Authentication

By ATCC (webiste suggest STR profiling, karyotyping)

Mycoplasma contamination

The cell line was not tested for mycoplasm contamination after being received from ATCC.

Commonly misidentified lines
(See ICLAC register)

none

Animals and other organisms

Policy information about studies involving animals; ARRIVE guidelines recommended for reporting animal research

Laboratory animals

Mus musculus: The Gpr156del strain (B6N(Cg)-Gpr156tm1.1(KOMP)Vlcg/J; MGI:5608696) was produced by the Knockout Mouse Project consortium (KOMP). The following published strains were used: Dapledel (C57BL/6J-Ccdc88ctm1(KOMP)Mbp; MGI:5141808), R26LSL-mycPTXa (Gt(ROSA)26Sorem1(ptxA)Btar; MGI:6163665) 8. R26LSL-Emx2 (RosaEmx2-Egfp) 27 was produced by Doris Wu at NIH/NIDCD. Vangl2Lp is (LPT/LeJ; MGI:1857642). References for the Cre lines are as follows: Atoh1-Cre (Tg(Atoh1-cre)1Bfri, MGI:3775845) 57, FoxG1-Cre (Foxg1tm1(cre)Skm; MGI:1932522) 58, Gfi1-Cre (Gfi1tm1(cre)Gan; MGI:4430834) 59. The Emx2del strain was generated in the C57BL/6J background with CRISPR/Cas9 to entirely delete the coding portion of Emx2 first exon. The following guide RNAs were used: 5'- TCGGCGCAGCATGTTTCAGC-3' and 5'- AGTTTCAGAACCAAGAACCC-3'. A founder mouse that contained the expected ~500bp deletion was identified by standard PCR. The strain was used for analysis after breeding with wild-type C57BL/6J animals for 2 generations to avoid potential unwanted genomic alterations. Wild-type animals were either C57BL/6J inbred, or C57BL/6 x FVB/NJ outbred. Primers used for animal and sample genotyping are indicated in Supplementary Table. Mice were maintained under standard housing with a 14 hour light/10 hour dark cycle, ambient temperature and normal humidity.

Danio rerio: Male and female breeding adults (3 months- 1.5 years) were used to generate the larvae used in the study. The larvae used in the study and shown in the figures were at 5 days post fertilization. At this age (5 dpf), sex cannot be predicted or determined, and therefore sex of the animal was not considered in our studies. For calcium imaging in lateral-line hair bundles, the previously described transgenic line was used: Tg(-6myo6b:GCaMP6s-CAAX)idc1Tg 41. An existing zebrafish mutant, gpr156sa34566 was obtained from the Zebrafish International Resource Center†. This mutant results in a stop codon in the last coding exon (aa 734/797). This allele was genotyped using standard PCR and sequencing and the following primers set: FWD 5'-CCTCCGCTGGACTGATAGAG-3' and REV-5'- GCGGTAGAAATCCTCGTCCT-3'. A CRISPR-Cas9 gpr156 zebrafish mutant (gpr156idc15 , denoted as gpr156exon2 in the figures) was generated using CRISPR-Cas9 technology as previously described 59. The second coding exon was targeted using the following guide: 5'-CAGGAGACAGAGACCGACTC (TGG)-3'. Founder fish were identified using fragment analysis of fluorescent PCR products 59. From these founder fish, a gpr156 mutant was identified that contained a 7 bp deletion 5'-AGCAGTGTGGAT--(GTCCAGA)-- GTCGGTCTCTGTCTCCTG-3'. This 7 bp deletion results in a predicted stop codon at the middle of the third coding exon – just prior to the second transmembrane domain of Gpr156 (aa 109/797). Genotyping of this CRISPR mutant, gpr156idc15 was accomplished using standard PCR and sequencing using the following primers: FWD 5'-ATTTTGCCGTTTGTCTGAATCT-3' and REV 5'-AATACAGCTCTTGCTCCTGCTC-3'.

Wild animals

none

Field-collected samples

none

Ethics oversight

All mouse work was reviewed for compliance and approved by the Animal Care and Use Committee of The Jackson Laboratory

(Animal Use Summary #14012). All zebrafish work was performed at the National Institutes of Health (NIH) and was approved by the Animal Use Committee at the NIH under animal study protocol #1362-13.

Note that full information on the approval of the study protocol must also be provided in the manuscript.
